# Accurate and Automated High-Coverage Identification of Chemically Cross-Linked Peptides with MaxLynx

**DOI:** 10.1021/acs.analchem.1c03688

**Published:** 2022-01-11

**Authors:** Şule Yılmaz, Florian Busch, Nagarjuna Nagaraj, Jürgen Cox

**Affiliations:** † Computational Systems Biochemistry Research Group, Max-Planck-Institute of Biochemistry, Am Klopferspitz 18, 82152 Martinsried, Germany; ‡ Bruker Daltonics GmbH & Co. KG, 28359 Bremen, Germany; § Department of Biological and Medical Psychology, University of Bergen, 5007 Bergen, Norway

## Abstract

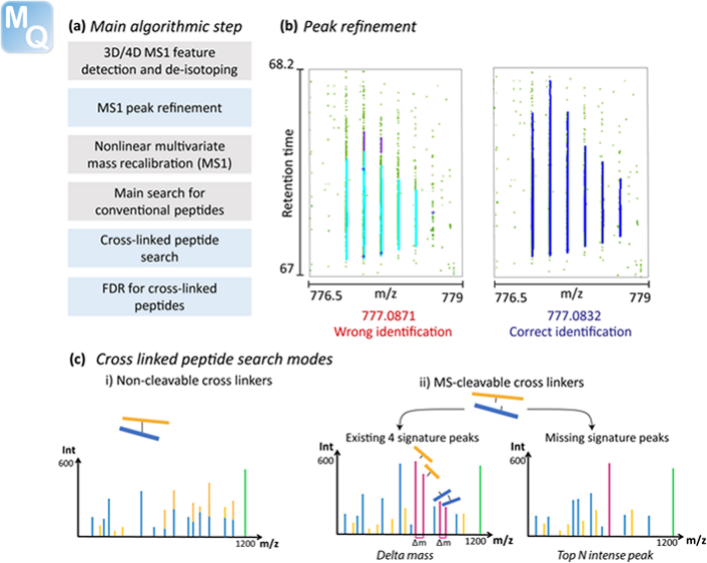

Cross-linking combined with mass spectrometry (XL-MS) provides a wealth of information about the three-dimensional (3D) structure of proteins and their interactions. We introduce MaxLynx, a novel computational proteomics workflow for XL-MS integrated into the MaxQuant environment. It is applicable to noncleavable and MS-cleavable cross-linkers. For both, we have generalized the Andromeda peptide database search engine to efficiently identify cross-linked peptides. For noncleavable peptides, we implemented a novel dipeptide Andromeda score, which is the basis for a computationally efficient *N*-squared search engine. Additionally, partial scores summarize the evidence for the two constituents of the dipeptide individually. A posterior error probability (PEP) based on total and partial scores is used to control false discovery rates (FDRs). For MS-cleavable cross-linkers, a score of signature peaks is combined with the conventional Andromeda score on the cleavage products. The MaxQuant 3D peak detection was improved to ensure more accurate determination of the monoisotopic peak of isotope patterns for heavy molecules, which cross-linked peptides typically are. A wide selection of filtering parameters can replace the manual filtering of identifications, which is often necessary when using other pipelines. On benchmark data sets of synthetic peptides, MaxLynx outperforms all other tested software on data for both types of cross-linkers and on a proteome-wide data set of cross-linked *Drosophila melanogaster* cell lysate. The workflow also supports ion mobility-enhanced MS data. MaxLynx runs on Windows and Linux, contains an interactive viewer for displaying annotated cross-linked spectra, and is freely available at https://www.maxquant.org/.

## Introduction

Chemical cross-linking combined with mass spectrometry (XL-MS) has undergone remarkable developments to become a promising complementary method for studying protein structure, conformation, and interactions.^1−4^ A typical protein cross-linking experiment starts with a formation of covalent bonds between spatially close residues in proteins or protein complexes through a cross-linker. A chemical cross-linker is typically specific to certain amino acids and its presence imposes a distance constraint.^5^ A cross-linked protein sample is then enzymatically digested and the resulting complex peptide mixture contains different types of products, including mono-, loop-, and cross-linked peptides.^6^ In fact, cross-linked peptides can be more diverse than just two peptides connected by a single linker due to the combination of multiple reactions, resulting in higher-order cross-linked peptides.^7,8^ Linear peptides, in which cross-linkers are not attached, are usually much more abundant in the sample than cross-linked products, and therefore enrichment of cross-linked peptides is generally required. The peptide mixture is analyzed using liquid chromatography coupled to tandem mass spectrometry (LC–MS/MS), and the resulting experimental MS/MS spectra are assigned to cross-linked peptides by using specialized algorithms.^4,9^ Eventually, the cross-linked peptide identifications are used to gain insights into protein structures or their interaction partners.^10,11^

XL-MS peptide identification algorithms can be subdivided based on the type of the supported cross-linker, which can be noncleavable or MS-cleavable. A noncleavable cross-linker remains intact during mass spectrometric analysis, whereas an MS-cleavable cross-linker fragments easily owing to a labile bond. The MS/MS spectra produced by these two approaches are qualitatively different. In both cases, the identification algorithm has to include different cross-link products in addition to single peptides in the search space, strongly increasing its complexity compared to conventional proteomics. In studies with noncleavable cross-linkers, any peptide can be linked to any other peptide, which causes a search space increase, known as the *N*-squared problem.^1,6^ Many programs such as StavroX^12^ and OpenPepXL^13^ use an exhaustive search in that space, which is computationally challenging, and therefore, noncleavable cross-linkers are commonly used for smaller sets of proteins or protein complexes. Some other algorithms have different approaches to tackle this search space problem. For example, xQuest^14^ detects isotopic pairs on the MS1 level with a mass shift observed by using the heavy- and light-labeled cross-linked peptides to select candidate peptides, and by that reduced the number of MS/MS spectra that need to be submitted to the search. Kojak^15^ has a two-pass approach: the first pass selects peptide candidates while allowing a differential modification mass, and the second pass creates cross-linked peptides from these candidates. In addition to this search space challenge, noncleavable cross-linked peptides can show an unequal distribution of the fragment ions from the two peptides.^6^ These two issues can be circumvented by using MS-cleavable cross-linkers,^16^ which enable even proteome-wide applications of XL-MS.^17,18^ The fragmentation of the labile bond of the MS-cleavable cross-linker produces single peptides with specific parts of the cross-linker attached. This results in the observation of distinctive signature ions.^16^ With the help of the signature peaks and the corresponding precursor, candidate cross-linked peptides are produced and then scored against the experimental spectrum. XlinkX^17^ and MeroX^19^ are two commonly used algorithms in MS-cleavable studies that use signature peaks in their algorithms, and XLinkProphet^20^ is an example of how machine learning approaches for validation can increase sensitivity.

MaxQuant^21^ is freely available computational proteomics software widely used in the community that supports diverse experimental designs and mass spectrometry platforms. Here, we describe the integration of novel tools and algorithms for the identification of cross-linked peptides in MaxQuant, collectively called MaxLynx. We evaluated MaxLynx on synthetic peptide data sets of cross-linked peptides obtained with the noncleavable and MS-cleavable approaches and compared its performance to results obtained with several other software packages. We found that at a 1% false discovery rate (FDR), MaxLynx outperformed many other software for both noncleavable and MS-cleavable data sets, with up to four times more cross-linked-peptide-to-spectrum matches (CSMs) and twice the number of unique cross-links. In addition, we performed a complex proteome-wide study and compared it to the published results from MeroX. We observed that MaxLynx again reported more CSMs along with more unique cross-links.

## Experimental Section

### Synthetic Benchmark Data Set

We downloaded the raw data from the data set using identifier PXD014337^22^ in the PRIDE repository,^23^ which contains cross-linked synthetic peptides linked by noncleavable and MS-cleavable cross-linkers and measured via LC–MS/MS. In total, 95 tryptic peptides from the *Streptococcus pyogenes* Cas9 protein were chemically synthesized. Each of these peptides contains one internal lysine residue for cross-linking. Both the peptides N- and C-termini were modified to prevent unwanted cross-linking reactions. These peptides were split into 12 groups, and cross-linking experiments were performed only within each group. These 12 samples were then mixed before introduction to LC–MS/MS (Orbitrap Q-Exactive HF-X). Disuccinimidyl suberate (DSS) was used to create noncleavable cross-linked peptides and these were measured as three technical replicates. Disuccinimidyl dibutyric urea (DSBU) and disuccinimidyl sulfoxide (DSSO) were used to create MS-cleavable data and measurements were performed with stepped higher-energy collision-induced dissociation (HCD) using an Orbitrap Q-Exactive HF-X instrument without technical replication for each data set.

### Proteome-Wide Benchmark Data Set

We downloaded the raw data from data set PXD012546^18^ in the PRIDE repository to evaluate the performance of MaxLynx in proteome-wide studies. Three biological replicates of *Drosophila melanogaster* (fruit fly) embryo extracts were cross-linked using DSBU and separated using size exclusion chromatography, resulting in 67 LC–MS/MS runs. The samples were measured with stepped HCD using an Orbitrap Q-Exactive Plus mass spectrometer.^18^

### timsTOF Pro BSA Data Set

Bovine serum albumin (BSA) (GERBU Biotechnik GmbH, #1062) was dissolved in 50 mM phosphate-buffered saline (PBS) pH 7.0 and the protein solution was transferred into Amicon Ultra 0.5 mL centrifugal filters (10 kDa NMWCO). After several rounds of dilution with 50 mM PBS pH 7.0 and reconcentration by centrifugation to remove potential interfering small molecules, the protein concentration was adjusted to 10 μM. Proteins were cross-linked at a molar ratio of cross-linker to protein of 25:1 by the addition of 1 μL of 50 mM DSSO (ThermoFisher Scientific, #A33545) and 50 mM DSBU (Bruker Daltonics, #1881355) into DSMO, respectively. After an overnight reaction at 4 °C, the reactions were quenched by the addition of 100 μL of 100 mM Tris/HCl pH 7.5. Proteins were denatured by buffer-exchange to 50 mM ammonium bicarbonate with 8 M urea, and reduced/alkylated by incubation with DTT (5 mM) for 30 min and iodoacetamide (15 mM) for 20 min at room temperature. Proteins were buffer-exchanged to 50 mM ABC and digested with 4 μg of Trypsin Gold (Mass Spectrometry Grade, Promega, #V5280) for 1 h at 37 °C. The flowthrough after centrifugation was combined with the flow through from an additional wash of the filters with 200 μL of water with 0.1% formic acid. After evaporation of solvent by SpeedVac vacuum concentration, peptides were resuspended in water with 0.1% formic acid. LC–MS/MS. A 200 ng of digested sample was analyzed using a nanoElute coupled to a timsTOF Pro mass spectrometer (Bruker Daltonics). Peptides were separated on an Aurora C18 column (25 cm × 75 μm i.d., 1.6 μm particle size, Ionoptics, AUR2-25075C18A-CSI) at a flow rate of 0.4 μL/min at 50 °C. The following gradient was used: within 60 min from 2 to 17% B, within 30 min from 17 to 25% B, within 10 min from 25 to 37% B, within 10 min from 37 to 80% B, and isocratic at 80% B for 10 min. Solvent A was water with 0.1% formic acid, and solvent B was acetonitrile with 0.1% formic acid. Mass spectra were recorded from *m*/*z* 100 to 1700 and showed inverse reduced mobility of 0.6–1.52 V s/cm^2^. Charge states for PASEF were set to 3–5 and selected ions were fragmented by TIMS stepping with two 1/*K*_0_-dependent collision energies (collision energies linearly interpolated between 0.85 and 1.2 V s/cm^2^ to 25–55 and 30–70 eV). 10 PASEF MS/MS scans were triggered per cycle (2.23 s).

### Data Analysis

#### Synthetic Benchmark Data Set

For the synthetic cross-linked peptide library data sets (PXD014337), the common search settings were appended to the given table given by Beveridge and coworkers^22^ (see Tables S1 and S2). The search databases used were Cas9 plus 10 proteins and Cas9 plus 116 cRAP contaminant proteins for the noncleavable and MS-cleavable data sets, respectively. These default MaxQuant search settings were changed: “include contaminants” was disabled, and including any loss and also higher charge states in the MS/MS analyzer section was also disabled. The new option called “peak refinement” was enabled. No cross-link-specific filtering was enabled, which is the minimum score for cross-linked peptides, the minimum score for other cross-linked products, and the minimum number of fragment ions from each peptide. The minimum partial score remained at the default value of 10 for both the noncleavable and the MS-cleavable data sets. For all data sets, the MaxQuant results were kept at the CSM-FDR of 1% for further comparison. Because of the peptide level experimental design,^22^ the software performance was evaluated without relying on protein structures: from a given list of CSMs, a CSM was considered to be correct when cross-linked peptides belonged to the same peptide group, otherwise it was assigned as incorrect.

#### Proteome-Wide Benchmark Data Set

The following settings were changed from MaxQuant default values: a minimum peptide length of five and a maximum peptide mass of 8000 Da was used. No contaminants were added. Only cross-linked peptides (interpeptides with a single cross-link modification) were considered to be able to fairly compare against the published results. The selected enzyme was trypsin, with four missed cleavages. The peptide and fragment ion tolerances were 5 and 15 ppm, respectively. No high charges or losses were allowed for the FTMS MS/MS analyzer settings. Carbamidomethylation of cysteine was chosen as a fixed modification, and oxidation of methionine and acetylation of protein N-terminus were chosen as a variable modification, allowing two modifications per peptide. The search database contained the identified Drosophila proteins (ID with 9535 protein entries), as recommended by Götze and coworkers.^18^ The MaxLynx results were compared at 1% FDR.

#### timsTOF Pro BSA Data Set

The search database contains the BSA protein plus *Pyrococcus furiosus* (PFU) proteins (502 reviewed protein sequences, downloaded on March 8th, 2021 from UniProtKB^24^). Using PFU proteins as an entrapment database has been previously proven as a good way to validate proteomics results.^25^ In our analysis, we used this approach to evaluate the performance of MaxLynx. A CSM including only BSA proteins was likely to be correct, whereas any CSM containing PFU proteins was incorrect. This additional criterion further allowed us to validate our cross-link identifications independent of protein structure information. The search settings were as follows. Oxidation of methionine and carbamidomethylation of cysteine are taken as variable and fixed modification, respectively, with a maximum of one variable modification per peptides. Trypsin is selected as an enzyme with a maximum of three missed cleavages. The cross-linkers are either DSSO or DSBU (both as heterobifunctional as a lysine residue can be linked to lysine, serine, threonine, and tyrosine residues) with mono- and cross-linked peptide options. The minimum peptide length was six, and the maximum peptide mass was 6000 Da. Contaminants were not included. The default MaxQuant maximum charge was changed from 4 to 6 in the Bruker TIMS instrument settings.

#### Reprocessing a Medium-Size Complex Data Set

We reprocessed a publicly available data set of a medium-sized protein complex (PXD013947).^26^ This study revealed the structural changes of human transcription factor IIH while switching from a transcription to a repair factor. The search database contains nine protein sequences. We ran MaxLynx and recent pLink version 2.3.9. The common search settings were as follows: oxidation of methionine and carbamidomethylation of cysteine were taken as variable and fixed modifications, respectively. The cross-linker was BS3. The minimum peptide length was six, and the maximum peptide mass was 6000 Da. Trypsin was selected as the enzyme with maximally three missed cleavages. Precursor and fragment tolerances were 10 and 20 ppm, respectively. For the MaxLynx-specific search settings, higher charges and neutral losses were disabled. Results were compared at a separate FDR = 1%.

### Software Availability, Requirements, and Usage

MaxLynx is freely available at www.maxquant.org as a part of MaxQuant, the cross-link search module was written in the C# programming language and integrated into the existing MaxQuant workflow.^21^ The program can run as a graphical user interface tool on Windows and also on the command line on both Windows and Linux operating systems.

### Data Availability

The MaxLynx processed results including output tables, search settings (mqpar files), and MaxLynx software (MaxQuant version 2.0.4 RC1) were deposited to PRIDE (http://proteomecentral.proteomexchange.org). The re-analyzed data sets PXD014337 and PXD012546 are available as PXD027159 (username: reviewer_pxd027159@ebi.ac.uk and password: Mp4ZPwhA) and PXD027188 (username: reviewer_pxd027188@ebi.ac.uk and password: 1z59ewcF), respectively. The timsTOF Pro BSA data set is available as PXD027161 (username: reviewer_pxd027161@ebi.ac.uk and password: Xwq80AV2). The reprocessed data set of PXD013947 is available as PXD030578 (username: reviewer_pxd030578@ebi.ac.uk and password: Gzr3RE3J).

## Results and Discussion

### MaxLynx Workflow

**Figure 1:**
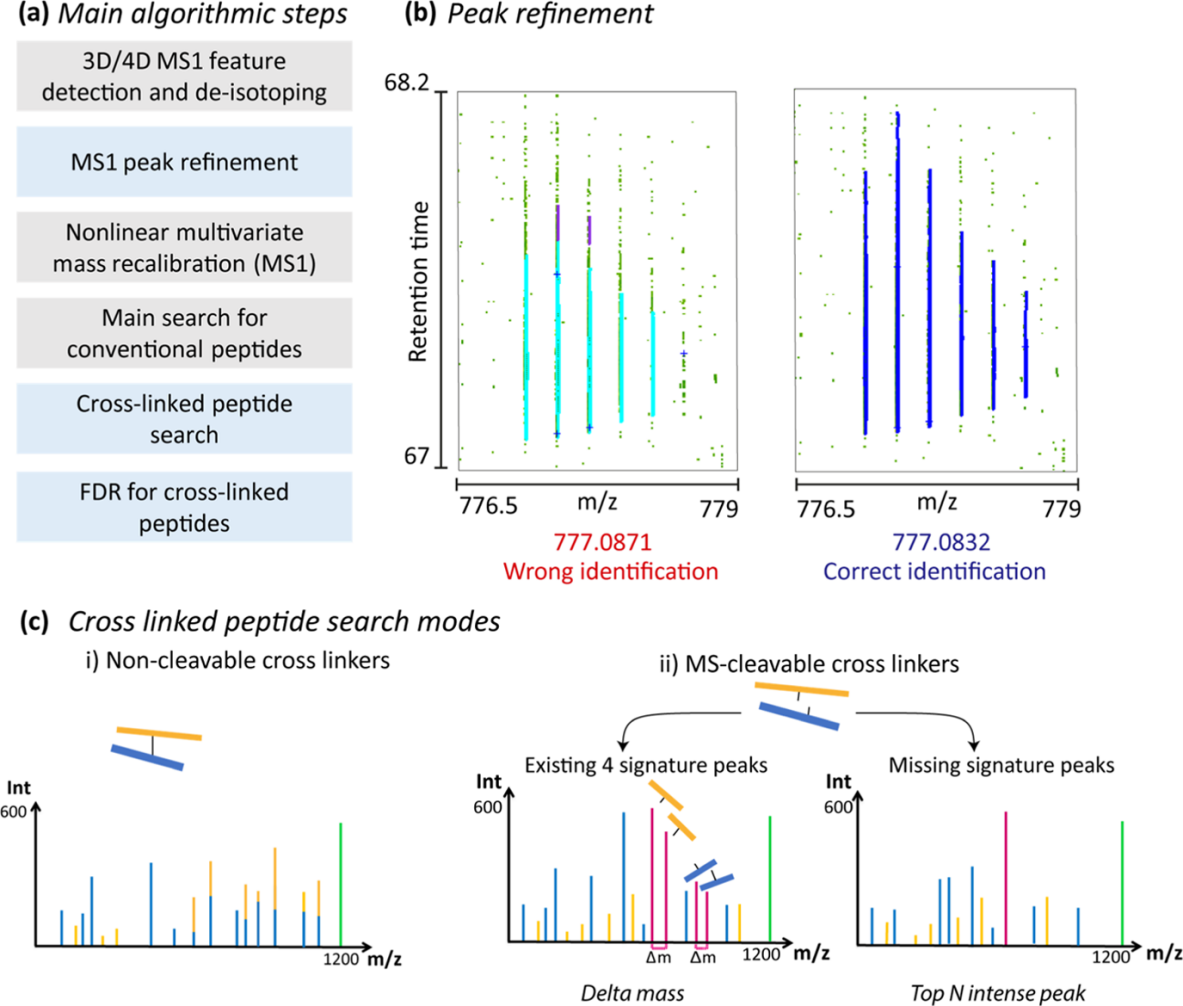
Computational workflow of MaxLynx. (a) Schematic simplified block diagram of the main algorithmic steps involved in MaxLynx. Steps in gray are unchanged from the MaxQuant workflow for regular peptides, while blue steps are newly developed for the cross-linking search. (b) Peak refinement is a computational step which was added after the peak detection with the aim of “repairing” peaks typically of heavy mass that are not well defined due to noise. (c) Depending on whether the linker that has been applied is MS-cleavable, one of two search engines is employed to query the measured MS/MS spectra.

Much of the MaxQuant workflow for conventional peptides is used in MaxLynx as well (Figure 1a), for instance, the detection and de-isotoping of features in the MS1 data. These are usually three-dimensional (3D) objects spanned by *m*/*z*, retention time, and signal intensity. We also support ion mobility-enhanced data,^27^ generated from the timsTOF Pro instrument, in which case the MS1 features become four-dimensional. The novel peak refinement feature (Figure 1b) is executed after the peak detection for data without ion mobility and assembles peaks that were not properly put together due to noise, which may happen for peptides with higher masses. These not well-assembled peaks lead to wrong assignments of the monoisotopic peak to the isotope pattern,^28^ which in turn hinders their identification. This problem is strongly reduced by peak refinement. In peak refinement, 3D peaks are first assembled into putative clusters by assembling peaks at the same *m*/*z* value and that are separated in retention time, whenever there is a third 3D peak in an *m*/*z* distance that corresponds to a neighboring peak in an isotope pattern that would cover the gap in retention time. Peaks in these clusters are then joined in retention time direction whenever the cluster spans at least three isotopic peaks.

The conventional Andromeda search engine^29^ is used to identify linear peptides and also to perform nonlinear mass recalibration. For the identification of cross-linked peptides, one of two specialized search engines is used (Figure 1c), depending on whether the cross-linker is MS-cleavable. Finally, a module for applying a desired FDR to the level of CSMs based on a posterior error probability (PEP) calculation is included. A list of all the detailed steps involved in MaxLynx is shown in Figure S1. A user’s guide on how to use MaxLynx can be found in the Supporting Information. Any bifunctional noncleavable or MS-cleavable cross-linker is configurable in the user interface (Figure S2).

**Figure 2:**
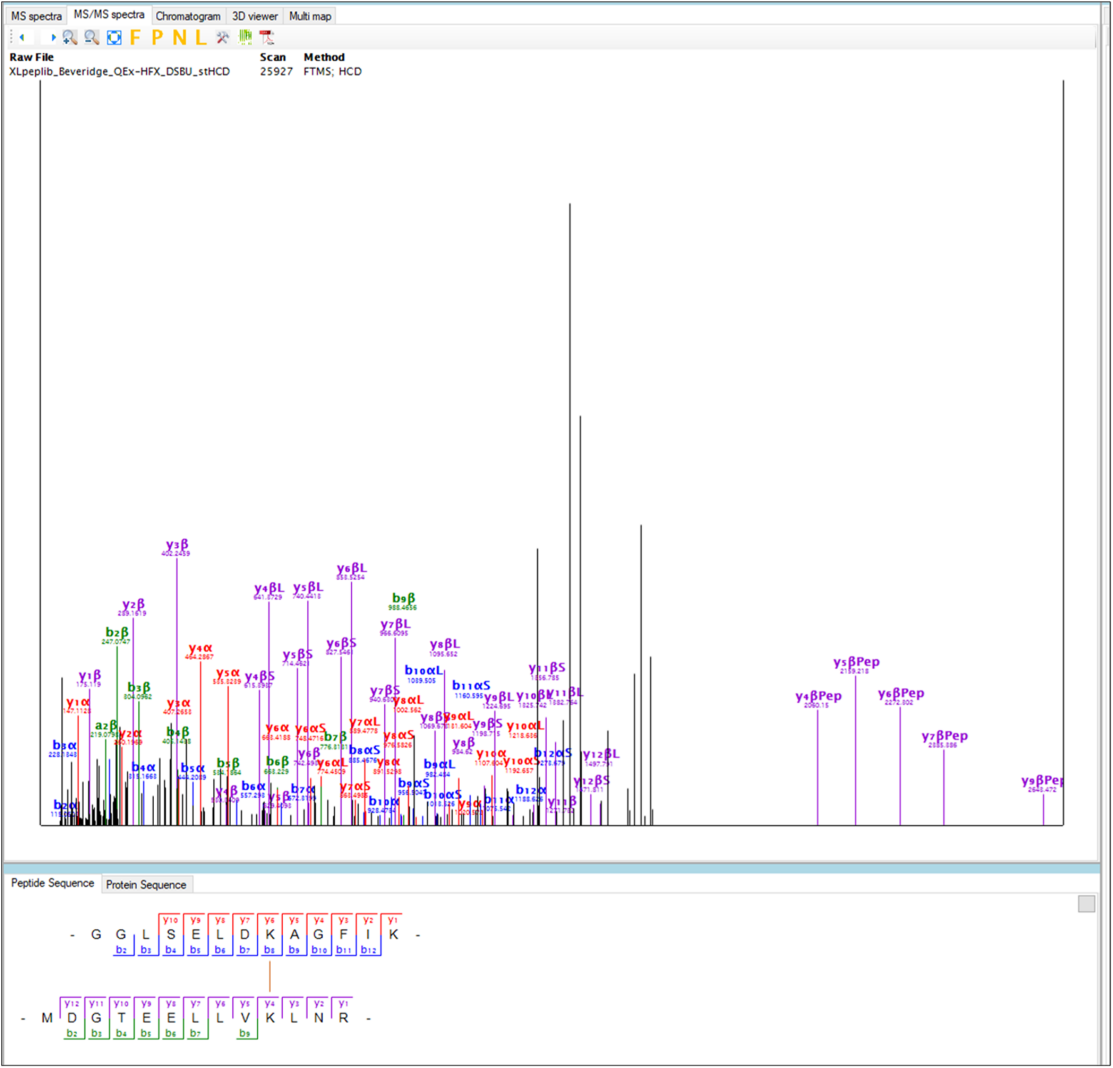
Visualization of identified cross-linked peptide. A screenshot of the MS/MS viewer in MaxQuant with annotation for fragments resulting from cross-linked peptides.

Cross-linked peptide identification results can be further inspected through MaxQuant Viewer interactively when a MaxLynx run is successfully completed (Figure 2). All defined cross-link products such as monolinks or dipeptides can be viewed, also for ion mobility-enhanced data sets. For dipeptides, the shorter (beta) and longer (alpha) peptides are color coded the same on both the “spectrum” and “peptide sequence” panels. On the “spectrum panel”, ion types, their numbers and their *m*/*z* values are provided. On the “peptide sequence” panel, a cross-linker is shown in red between two peptides (see Supporting Information).

### Noncleavable Cross-Linked Peptide Search

MaxLynx generates a complete search space for noncleavable cross-linked peptides and performs an exhaustive search in it. The first step is to generate in silico peptides depending on the Andromeda search settings, and then the search space is constructed by combining all putative peptides. The cross-link search space includes single peptides and different cross-linking products (dipeptides, monolinked, or loop-linked peptides) in case a peptide has a target residue for the cross-linker of choice. A user can define cross-linking products on the new group-specific parameter panel called “cross-links” (see Supporting Information and Figure S3). Monolinked peptides are added to the MaxLynx searches because two adjacent peptides can mimic a monolinked peptide or dipeptide because they have the same precursor mass.^30^ In case there are two or more target resides on a peptide, loop-linked peptides are created. In addition to these three cross-linked peptide products, single peptides without cross-linker modifications are added to the search space as well by default. Furthermore, MaxLynx can consider higher-order cross-linked peptides, which are the combination of different single cross-link modifications (e.g., an interpeptide product has a monolink, also known as type 2, 1^7^). Note that the complexity of the search space will be increased even more when these cross-linking products are added, and hence the results will include more false positives. Therefore, many other algorithms do not consider such multiple modifications. The cross-linking products, which are mono-, loop-, and cross-linked, and if selected, higher-order cross-linked peptides, are internally written to temporary files with their masses, the number of links, the linked sites, and peptide level information. Then, for each cross-link product, variable modifications are added to their masses, and then a single index is created.^29^

The second main step after the cross-link space construction is the MS/MS cross-link search. This step is integrated into the established Andromeda scoring workflow. The precursor mass of an experimental MS/MS spectrum is compared against indexed masses, and when an indexed mass is equal to the experimental precursor mass within a certain tolerance, a theoretical spectrum is generated. It is important to highlight the main difference compared to ordinary peptide searches: a theoretical spectrum for cross-linked peptides has theoretical fragment ions from both peptides and ions from the residue contributing to cross-linking have a corresponding mass shift.

### MS-Cleavable Cross-Linked Peptide Search

Another approach to studying cross-linked peptides is based on using MS-cleavable cross-linkers. During MS/MS analysis, the cross-linker is cleaved and this cleavage results in two peptides with partial cross-linkers attached (Figure 1c). The longer of the two peptides is denoted with the Greek letter alpha and the shorter with beta. The structures of the various MS-cleavable cross-linker molecules vary, but typically they have two labile bonds, so the cleavage commonly results in peptides containing either a shorter or a longer piece of the cross-linker, and this produces in the MS/MS spectrum a characteristic doublet peak signal per peptide with a specific mass difference. With the help of the doublet signals, the masses of the two peptides can be determined individually, and cross-linked peptides are identified based on this knowledge. These characteristic doublet peak signals are called signature peaks because signature peaks are specific to an MS-cleavable cross-linker in use. In MaxLynx, we consecutively apply three approaches to detect signature peaks, the strict mass difference approach, the top intensities approach, and finally a second round of the mass difference approach with relaxed criteria. The strict mass difference approach depends on observing mass differences between the long and short versions of the remainder of the cleaved cross-linker on the same peptide for both pairs of peaks in the MS/MS spectrum. The top intensities approach checks for the most intense peaks in the MS/MS spectrum if they can be interpreted as one of the signature peaks without requiring the other signature peaks to be present. In the mass difference approach with relaxed criteria, only one pair of signature peaks is required. All three approaches work on the MaxQuant processed version of the MS/MS spectra for which peaks have been de-isotoped and higher charge states of fragment ions have been transformed into charge one.^29^

In the strict mass difference approach, we aim to find all four signature peaks with two pairs having the expected mass difference between the long and short linker residual, denoted as Δ*m*. For that purpose, we loop through all peaks in the MS/MS spectrum that are larger than a user-definable minimal mass with the hypothesis that it is the β-peptide with the shorter linker residual (βs). Then, we check for the presence of the three corresponding remaining signature peaks, which are the β-peptide with the longer linker residual (βl) and both versions of the longer peptide (αs and αl) whose masses are given by
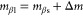

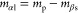

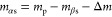
where *m*_p_ is the measured precursor mass of the whole dipeptide. In case all the four peaks are found with the given precursor mass tolerance, they are accepted as the signature peaks. Based on this, all theoretical spectra are constructed with y- and b-ion series for linear α- and β-peptides whose masses are compatible within the mass tolerance.

A weakness of the mass difference approach is that four signature peaks must be observed. However, it is not always the case that all of these are present in the spectrum.^31^ Furthermore, there could be homodimeric peptides, meaning that the peptide is linked to a peptide with the same sequence and therefore only two signature peaks exist. Indeed, a recent study^22^ showed that XlinkX,^17^ an algorithm using the mass difference strategy, did not report any such homodimeric peptides. To overcome this problem, we implemented a second step to find signature peaks in a less stringent way by choosing candidate peaks based on their intensities.^31^ This is performed whenever the mass difference approach does not find a solution. The assumption here is that the signature peaks are among the most intense peaks.^31^ We go through the top *n* most intense peaks in the MS/MS spectrum, where *n* = 3 by default, which can be changed by the user. For each of the top peaks, it is hypothesized that they are either carrying the longer or the shorter linker residue. Knowing the precursor mass, both hypotheses lead to masses for the α- and β-peptides. If both the calculated α- and β-peptide masses are heavier than the given minimum peptide mass, a theoretical combined spectrum is submitted to the database search. We observed that the signature peaks could sometimes have an ammonia (NH_3_) loss. Therefore, we extended the assumption above by considering that the top intense peaks can also have such a loss. This is expected, especially for fragments containing lysine residues.^29^ Based on the α- and β-peptides found, cross-linked peptide products are in silico constructed, followed by theoretical MS/MS spectrum generation and scoring in the Andromeda search.^29^ In case neither of the two approaches described finds a candidate explanation for an MS/MS spectrum, we perform another round with the mass difference approach, in which it is sufficient if only one peak pair with the characteristic mass difference is found.

### FDR Control

The FDR control in MaxLynx is based on the target-decoy strategy. A cross-link search results in a list of CSMs which can be split into three cross-link product groups: (i) dipeptides, in which two peptides are linked, (ii) single peptides with attached linker molecules, which are mono- and loop-linked peptides, and (iii) single ordinary peptides that no cross-linker is attached to. Dipeptides can be target–target (TT), target–decoy (TD), or decoy–decoy (DD) cross-linked dipeptides.

The PEP is the likelihood of a CSM being wrongly identified at a given Andromeda score and additional selected peptide properties. The PEP calculation using MaxQuant^21^ includes the logarithm of an identified peptide length. For MaxLynx, however, we modified our implementation to calculate the PEP for dipeptides, as a consequence of the coexisting two peptides. An unequal fragmentation is problematic for dipeptides^32^ and has a negative effect on scoring algorithms because search engines can still assign a good score despite very little or no evidence from one peptide but good fragmentation on its paired peptide. To overcome this issue, we decided to use the minimal partial score instead of peptide length in our PEP calculation for dipeptides. The partial score is a version of the Andromeda score that is calculated using only ions from one peptide of the cross-linked dipeptide against a given experimental MS/MS spectrum. Every cross-linked dipeptide has two partial scores, an α and a β partial score, and out of these, the minimum is used for the PEP calculation. The PEP is subsequently corrected for the number of modifications, the precursor charge state, and the biggest number of missed cleavages from the peptides involved in dipeptides. Afterward, all CSMs are sorted based on their PEP scores in an ascending order, and the FDR is calculated as the number of false CSMs divided by the number of target CSMs. Here, false CSMs are any identification of TD and DD cross-linked dipeptides. Then the FDR is calculated as the ratio of these false CSMs (#TD plus #DD) divided by the number of target cross-linked dipeptides (#TT). MaxLynx offers the option to either perform separate FDR calculations for protein intra- and inter-cross-links or to treat them together, the effect of which has been studied in Figures S10–S12.

### Benchmarks on Synthetic Cross-Linked Peptides

We re-analyzed publicly available data sets in which synthetic peptides were cross-linked with the noncleavable cross-linker DSS and with the MS-cleavable cross-linkers DSSO and DSBU. Beveridge and coworkers^22^ benchmarked these data sets by using several existing software platforms. For the noncleavable cross-linker data pLink2,^33^ StavroX,^12^ Xi,^34^ and Kojak^15^ were benchmarked, whereas for the MS-cleavable cross-linker data MeroX^19^ and XlinkX^17^ were used. This data was also analyzed with OpenPepXL in their own publication.^13^ We compared the MaxLynx results to the results provided in these two studies in terms of the number of CSMs and unique cross-linking sites at CSM FDR = 1%.

**Figure 3:**
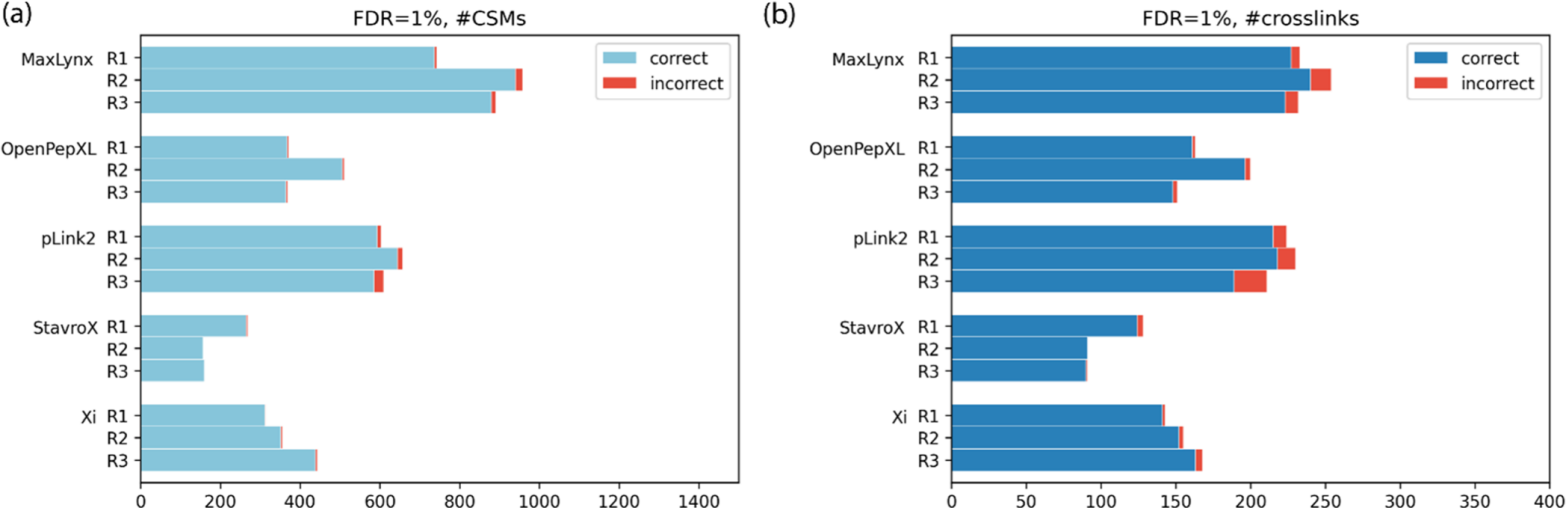
Comparison of MaxLynx against other cross-link search engines on the noncleavable cross-linker data set. (a) Shows the number of CSMs, (b) shows the number of unique cross-links at FDR = 1%. The results of OpenPepXL were obtained from Netz and coworkers,^13^ and the results of other software were taken from Beveridge and coworkers.^22^ Three replicates were shown as R1, R2, and R3. Note that the figure contains homo-multimeric cross-links in case that software produces them.

For the noncleavable cross-linker data set, MaxLynx reported the highest number of CSMs at FDR = 1% compared to the other algorithms, with 852 correct and 12 incorrect CSMs on average (Figure 3 and Tables S3 and S4). Moreover, MaxLynx reported the highest number of unique cross-links (on average 230). In noncleavable cross-linker search, the settings that influenced the number of identifications the most were related to the peak refinement option. When this option was disabled, the average number of correct CSMs dropped to 666 and the average number of correct cross-links to 199. This shows that the studies on the improvement of feature detection for heavier peptides have a significant improvement in cross-linked peptide identifications. We recommend the peak refinement option be on for cross-link searches.

**Figure 4:**
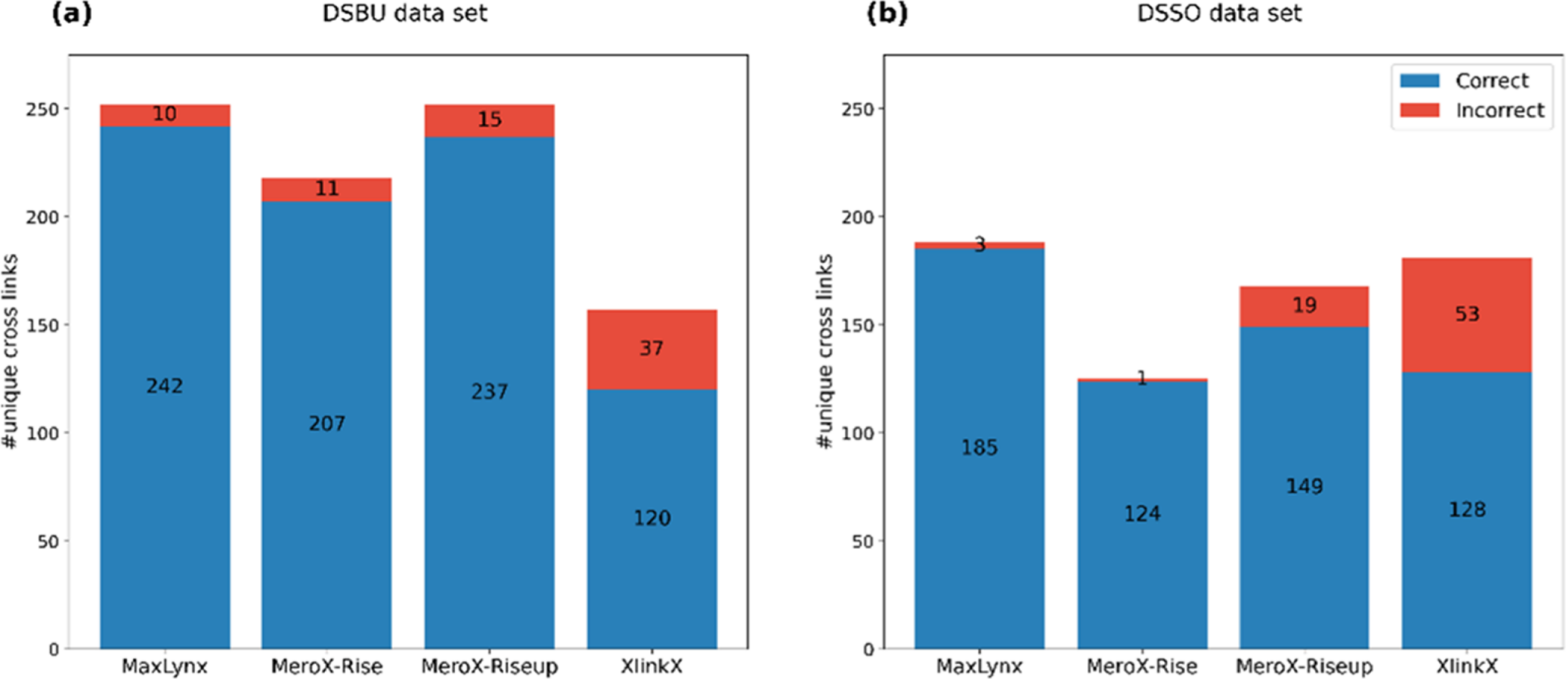
Comparison of MaxLynx against other cross-link search engines on the MS2-cleavable cross-linker data sets. (a) and (b) show the number of unique cross-links, respectively, at the DSBU and DSSO data sets at FDR = 1%. The results of the other search engines were obtained from Beveridge and coworkers.^22^ Note that the figure contains homomultimeric cross-links in case that software produces them.

On the MS-cleavable cross-linker data set, MaxLynx reported the highest number of correct unique cross-links for both the DSBU and DSSO data sets compared to the most of the other search engines^22^ (Figure 4 and Tables S5). For the DSBU data set, MaxLynx resulted in 242 correct and 10 incorrect unique cross-links at FDR = 1%. The MaxLynx results on the DSSO data set showed a similar pattern to the DSBU results. MaxLynx reported the highest number of correct cross-links at FDR = 1%, with 185 correct and 3 incorrect unique cross-links. One reason why MeroX performed better in terms of the identified number of cross-links was the ability to detect homomultimeric interpeptides,^22^ in which the peptide is linked to a peptide with the same sequence. For MaxLynx, the number of correct cross-links coming from the CSMs of homomultimeric interpeptides was 50 and 41 for the DSBU and DSSO, respectively. When these homomultimeric cross-links are ignored (192 and 144), MaxLynx still identifies a higher number of cross-links than is found with MeroX.

Furthermore, we have performed analysis to see the effect of using different MaxLynx parameter values (Supporting Information section: MaxLynx-specific parameter analysis and Tables S6–S14 and Figures S4–S12). We suggest using partial score = 10 and no additional score filtering and separating protein inter- and intra-cross-links for FDR calculation, in addition to disabling any high charge or losses for only FTMS analyzers. In addition, we tested FDR = 5% and we observed that this resulted in an increase in mostly incorrect identifications or decoys without any improvement in correct hits (Supporting Information section: MaxLynx-specific parameter analysis, Tables S15 and S16). In addition, we currently have a FDR control only on the CSM level but not the unique cross-link level and suggest using FDR = 1%.

### Benchmark on Proteome-Wide MS-Cleavable Cross-Linker Data

**Figure 5:**
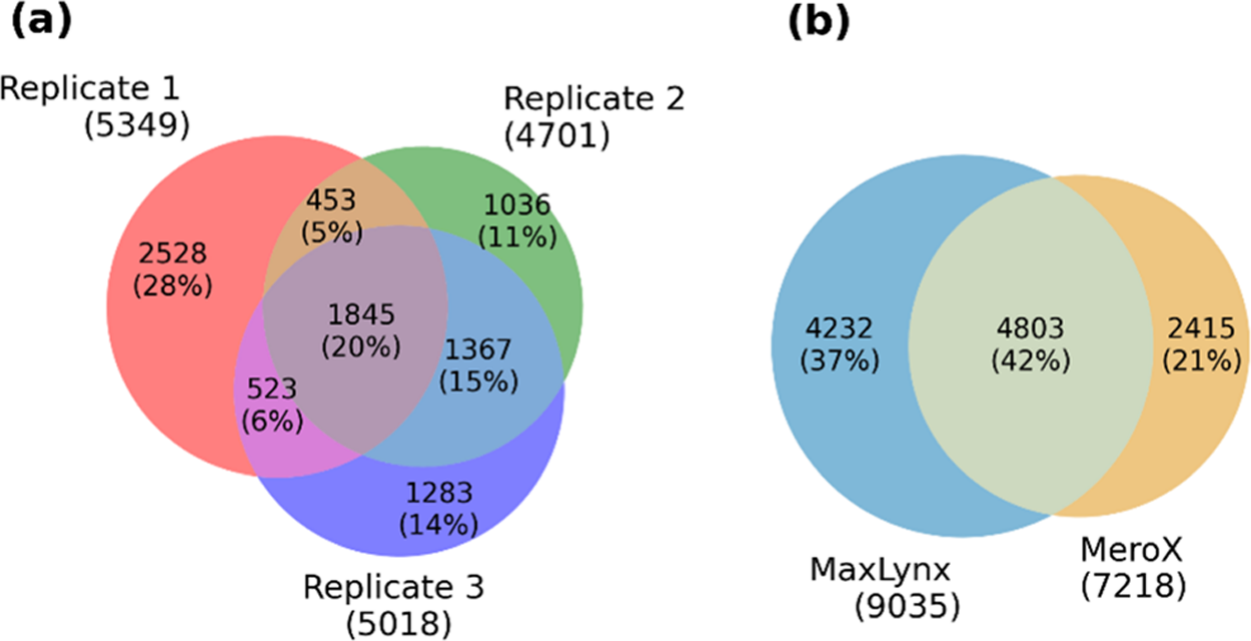
Overlap of unique cross-link sites for each replicate at the large-scale proteome-wide cross-link search. (a) Large-scale cross -ink experiment was performed in three replicates and the absolute number and the percentages are shown. (b) Total number of unique cross-links for MaxLynx and MeroX are compared.

Next, we evaluated the capability of MaxLynx to analyze large-scale, proteome-wide cross-linking data sets. For that purpose, we re-analyzed the PRIDE dataset PXD012546 of *D. melanogaster* embryo extracts cross-linked with DBSU and compared against the published results. At FDR = 1%, MaxLynx reported a total of 48,019 CSMs and 9035 unique cross-links, exceeding the originally reported number by MeroX, while using the same settings (Table 1). Although the reproducibility of identification results between the three replicates is with 20% (Figure 5a) found in all three rather low, it is higher than reported with Merox (overlap of 15%). As noted by Götze and coworkers,^18^ the reasons for this observation could be attributed to experimental and biological conditions. Next, we checked the number of unique cross-links overlapping between MaxLynx and MeroX software, and we observed that around 42% of all found unique cross-links were shared between these two (Figure 5b). We further checked the overlapping unique cross-links between the two software when a cross-link is found in at least 2/3 replicates and 3/3 replicates. One reason for observing more unique cross-links for MeroX and MaxLynx was due to the score cutoff from the FDR calculation (Figures S13 and S14). Despite this, it is clear that both search engines have search engine-specific cross-links. This can be explained by the differences in the rules for detection of signature peaks, and in particular how missing signature peaks are handled.

**Table 1:** Overview of the Results for the Proteome-Wide Study at FDR = 1%^a^

software	#CSMs	#unique cross-links	#unique intramolecule cross-links	#unique intermolecule cross-links
MeroX	29,931	7218	5110	2108
MaxLynx	48,019	9035	8662	373

a MeroX results were obtained from Götze and coworkers.^18^ Note that the number of unique cross-links and unique intramolecule cross-links were directly taken from their Supporting Information and their public PXD012546 data set.

### Ion Mobility-Enhanced Data

**Figure 6:**
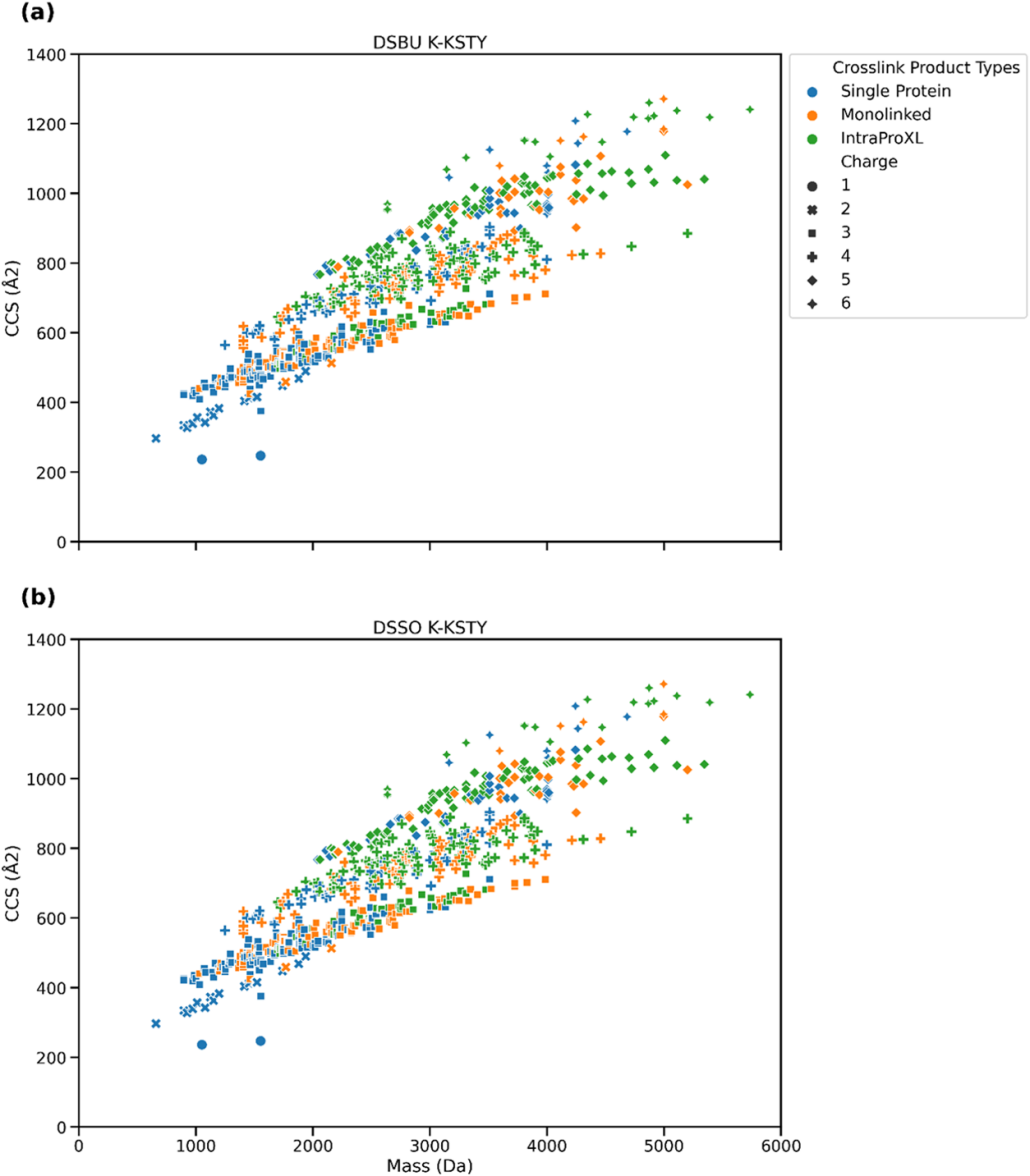
CCS values for timsTOF data set. CCS values are plotted against the molecule mass. (a) Results for DSBU. (b) Results for DSSO.

We have used PFU proteins as an entrapment database to evaluate the timsTOF Pro data set and the number of wrongly assigned CSMs was evaluated, that is, any CSMs that do not come from intra-BSA protein cross-links. The number of CSMs in total was 243 and 235 for DSBU and DSSO data sets, respectively, and within these only one and two CSMs were assigned to BSA–PFU protein cross-links, while no PFU–PFU links were found (Table 2). The number of incorrect CSMs is consistent with the CSM-FDR of 1% that was applied to the data. These results show that the search strategy by MaxLynx is sensitive while finding mostly correct hits. Next, we checked how the CCS values behave as a function of molecule mass with respect to the different cross-linked product types (Figure 6). We observe that cross-linked peptides tend to be have higher CCS values along with their high charge states and higher masses compared to linear peptides.

**Table 2:** Overview of the Results from the timsTOF Pro BSA Data Sets at FDR = 1%^a^

data set	#CSMs (all)	#CSMs for (BSA–BSA)	#unique cross-links (all)	#unique cross-links (BSA–BSA)
DSBU	243	243	127	127
DSSO	235	234	132	131

a The number of CSMs and their unique cross-links were reported for both DSBU and DSSO data sets including the CSMs to only BSA intraprotein cross-links, in addition to the number of unique cross-links.

### Reprocessing a Medium-Size Protein Complex Data Set

We re-analyzed a medium-sized complex data set (PXD013947) because using a synthetic benchmarking data set might not be sufficient to show MaxLynx’s performance on real-life data sets. With this real-life medium-size complex data set, the effectiveness of trypsin digestion will not be questionable while the peptides in the synthetic data sets were designed to contain only one mis-cleavage. The results demonstrate that MaxLynx performs well here as well. The number of CSMs was 2542 and 2335, and the number of unique cross-links was 315 and 287 for MaxLynx and pLink2, respectively. From these unique cross-links, MaxLynx reported 120 inter-protein cross-links, whereas pLink reported 94 (Table S17). The overlap between the unique cross-links is 60% (Figure S15). We then mapped MaxLynx results onto its 3D structure (PDB 6RO4). 109 unique cross-links could be mapped to the structure and 100 of these were within the cross-linker distance for BS3 (30 Å) (Figure S16). 1

## Conclusions

MaxLynx is a new computational workflow for XL-MS that is integrated into MaxQuant software. Here, we showed that MaxLynx outperforms benchmarked software for both noncleavable and MS-cleavable cross-linked peptide data sets at FDR = 1%. It works well for data with an ion mobility dimension as well. The success of the MaxLynx was also owing to the peak improvement of heavier peptides such as dipeptides. The percentage overlap of cross-links between the replicates is not yet ideal, but this may be overcome by better acquisition strategies and further improvements, such as introducing match-between-runs for cross-linked peptides and applying data-independent acquisition for such samples.

## Supporting Information Available

Step by step guideline on how to set up MaxLynx and the extended search settings along with the detailed results for the synthetic cross-linked peptides data sets and timsTOF and re-analysis of PXD013947 (PDF)
