# Supplementary material for: Accurate and Automated High-Coverage Identification of Chemically Cross-Linked Peptides with MaxLynx
Source: Anal Chem. 2022 Jan 11;94(3):1608–17. doi: 10.1021/acs.analchem.1c03688 (PMC8792900; doi:10.1021/acs.analchem.1c03688)
Supplement: Supplementary file 1 — ac1c03688_si_001.pdf [file ac1c03688_si_001.pdf]

SUPPORTING INFORMATION

Accurate and automated high-coverage identification of chemically cross-linked peptides with MaxLynx

Şule Yılmaz [1]*, Florian Busch [2], Nagarjuna Nagaraj [2], Jürgen Cox [1,3]*

[1] Computational Systems Biochemistry, Max-Planck-Institute of Biochemistry, Am Klopferspitz 18, 82152 Martinsried, Germany

[2] Bruker Daltonics GmbH & Co. KG, Bremen, Germany

[3] Department of Biological and Medical Psychology, University of Bergen, Bergen, Norway

*Correspondence: cox@biochem.mpg.de, yilmaz@biochem.mpg.de

TABLE OF CONTENTS

Contents

A user guide on how to run MaxLynx 4

1. Load your raw files and set your experiment design. 4

2. Set your MaxLynx parameters on the *Cross links* group-specific parameter tab. 5

2. a. Enable non-cleavable cross link search. 6

2. b. Enable MS-cleavable cross link search on MS2 level. 6

2. c. Configure your cross linkers. 7

4. Turn on Peak refinement. 8

5. Set up protein sequence related information. 8

6. Set your FDR values. 9

7. Disable losses and high charges for FTMS MS/MS analyser. 9

8. Note for Bruker TIMS instruments. 10

9. Inspect your results. 11

Supplementary Figure S1. 12

Supplementary Figure S2. 13

Supplementary Figure S3. 14

Supplementary Table S1. 17

Supplementary Table S2. 18

Supplementary Table S3. 19

Supplementary Table S4. 19

Supplementary Table S5. 20

MaxLynx-specific parameter analysis: 21

a. Combination with total-score and partial score values 21

Supplementary Figure S4. 22

Supplementary Figure S5. 22

Supplementary Figure S6. 23

Supplementary Figure S7. 24

Supplementary Figure S8. 25

Supplementary Figure S9. 26

b. Effect on separating protein intra- and inter-crosslinks for FDR control 27

Supplementary Figure S10. 27

Supplementary Figure S11. 28

Supplementary Figure S12. 29

Supplementary Table S6. 30

c. Effect of high-charged theoretical peaks and neutral losses 31
Supplementary Table S7. 32
Supplementary Table S8. 33
Supplementary Table S9. 34
Supplementary Table S10. 35
Supplementary Table S11. 36
Supplementary Table S12. 37
Supplementary Table S13. 38
Supplementary Table S14. 39
d. Effect of increasing FDR to 5% 40
Supplementary Table S15. 40
Supplementary Table S16. 40
Re-analysis of PXD012546 41
Supplementary Figure S13. 41
Supplementary Figure S14. 41
Supplementary Table S17 42
Supplementary Figure S15. 42
Supplementary Figure S16. 43
Running times 44
Supplementary Table S18 44
Re-analysis of PXD013947 44
References 45

A user guide on how to run MaxLynx

MaxLynx was integrated into the MaxQuant environment. You can download MaxQuant from https://maxquant.org/maxquant/

You must make sure to install .NET Core 2.1 (not higher or not earlier releases) SDK x64 from https://dotnet.microsoft.com/download/dotnet/2.1

1. Load your raw files and set your experiment design.
Note that MaxLynx currently works on only one parameter group.

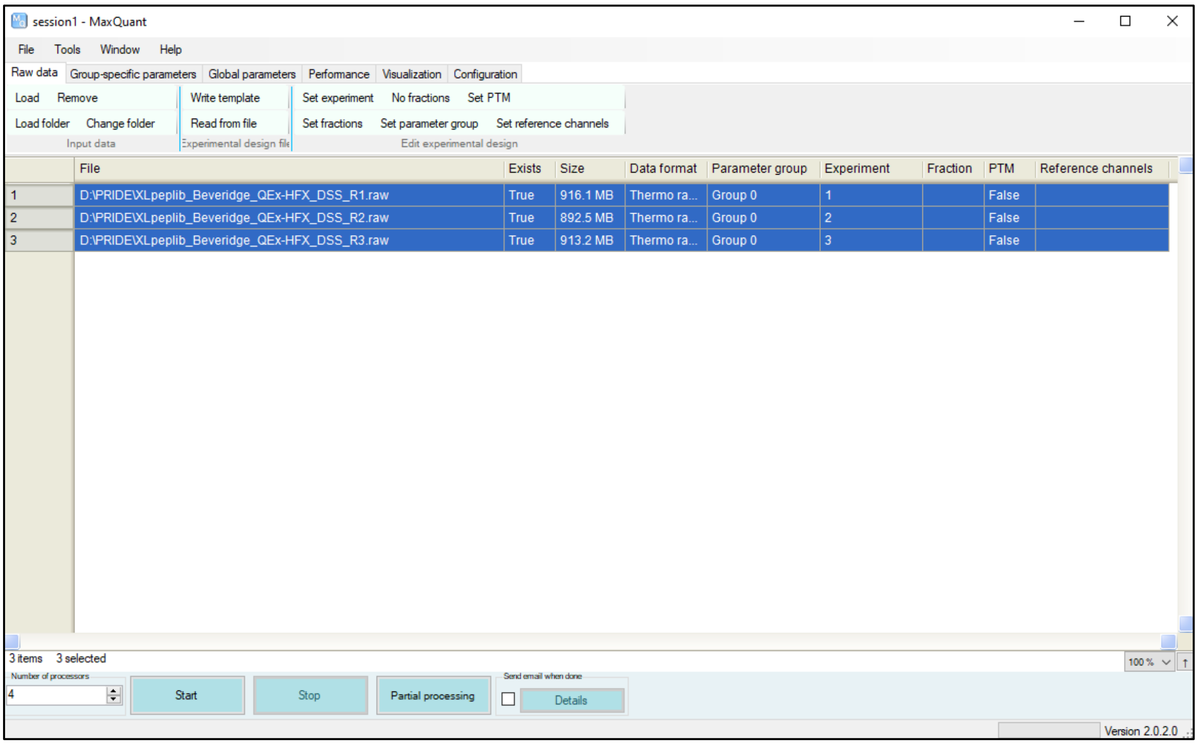

2. Set your MaxLynx parameters on the *Cross links* group-specific parameter tab.

This is a new MaxQuant group-specific parameter tab to set MaxLynx runs. It is important to choose the right cross linker type of your experiment.

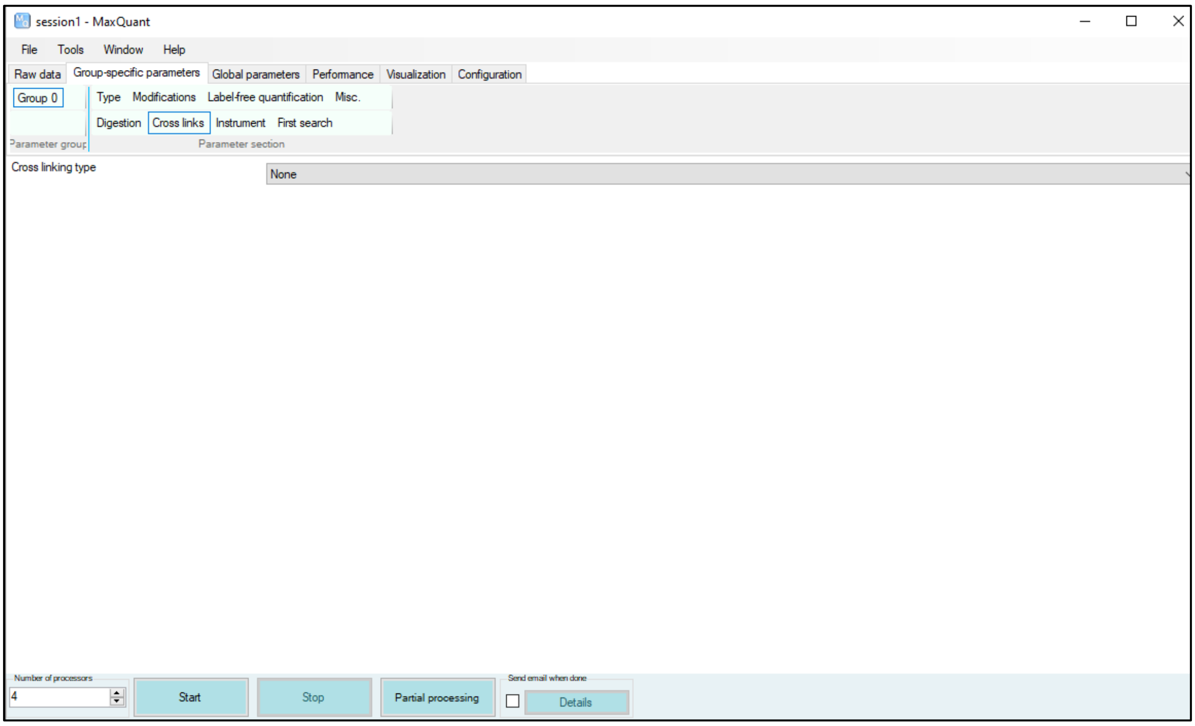

2. a. Enable non-cleavable cross link search.

Select by the *Cross linking type* as *Non-cleavable.*

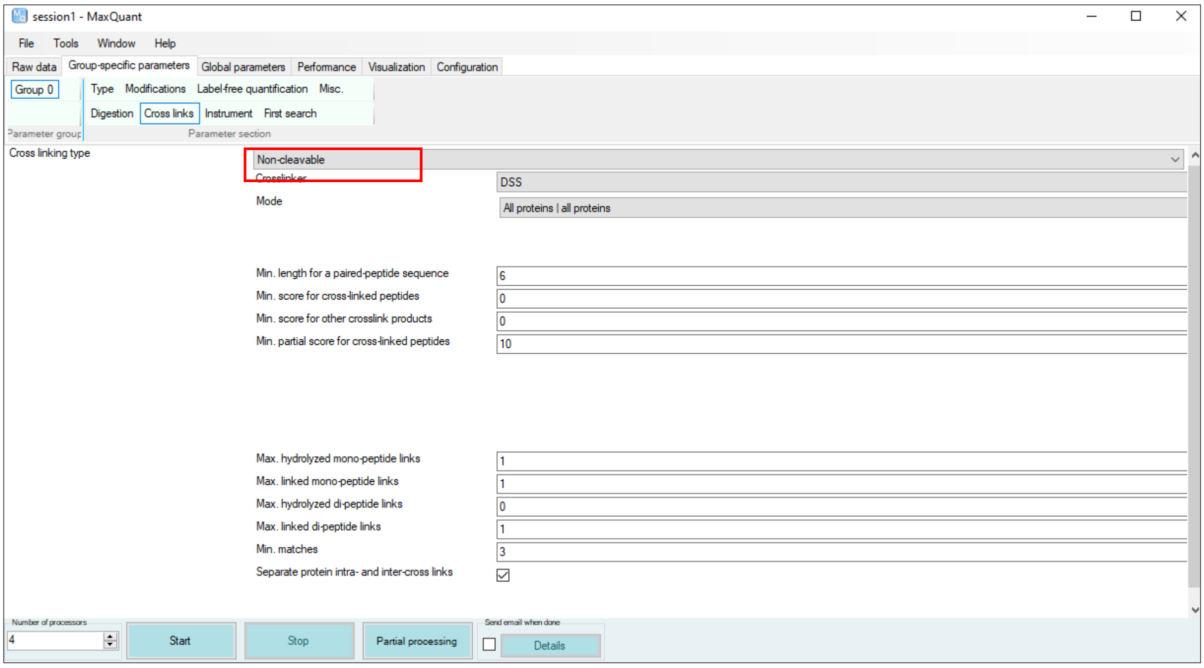

2. b. Enable MS-cleavable cross link search on MS2 level.

Select by the *Cross linking type* as *MS2-cleavable.*

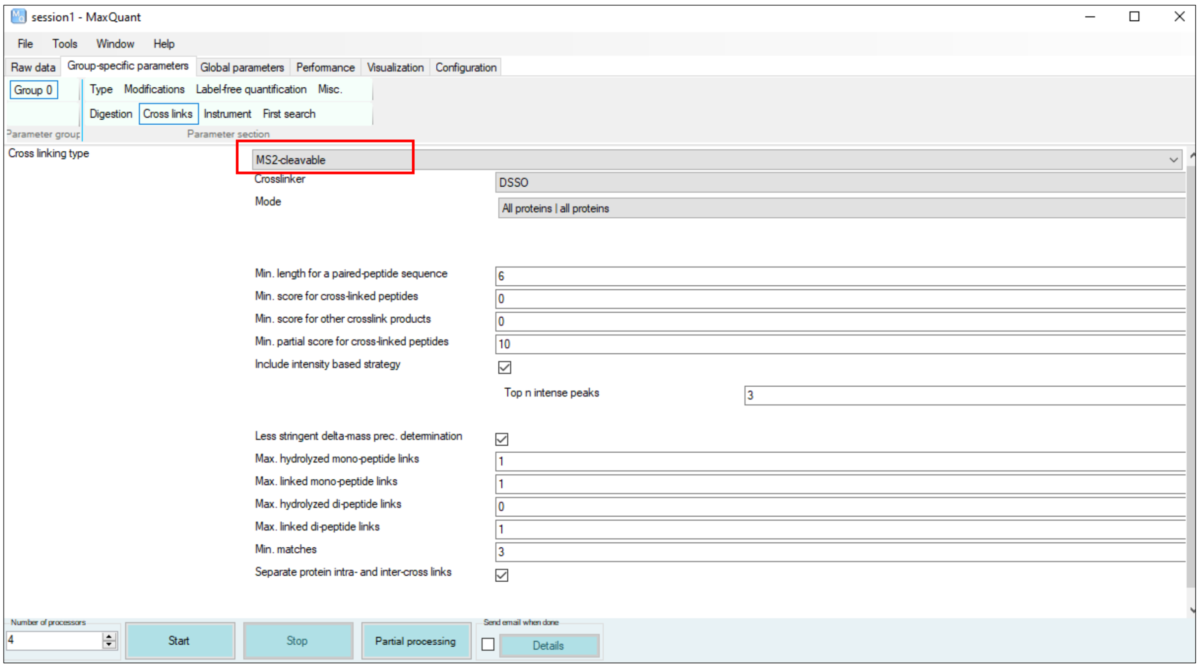

2. c. Configure your cross linkers.

You can configure any bi-functional cross linker through this new MaxQuant configuration tab called the *Crosslinks* under the *Configuration* panel. Provide a name and description of your cross linker. Add the compositions for cross link products, where two arms of a cross linker are attached to either mono- or di-peptides (*Linked composition*) and mono-link products, where only one arm of a cross linker is attached to one peptide (*Hydrolyzed composition)* along with the cross linker specificities. You can define *MS-cleavable cross linkers* by selecting the *MS-cleavable* box and then defining the compositions for the long and short parts of this cross linker after cleavage.

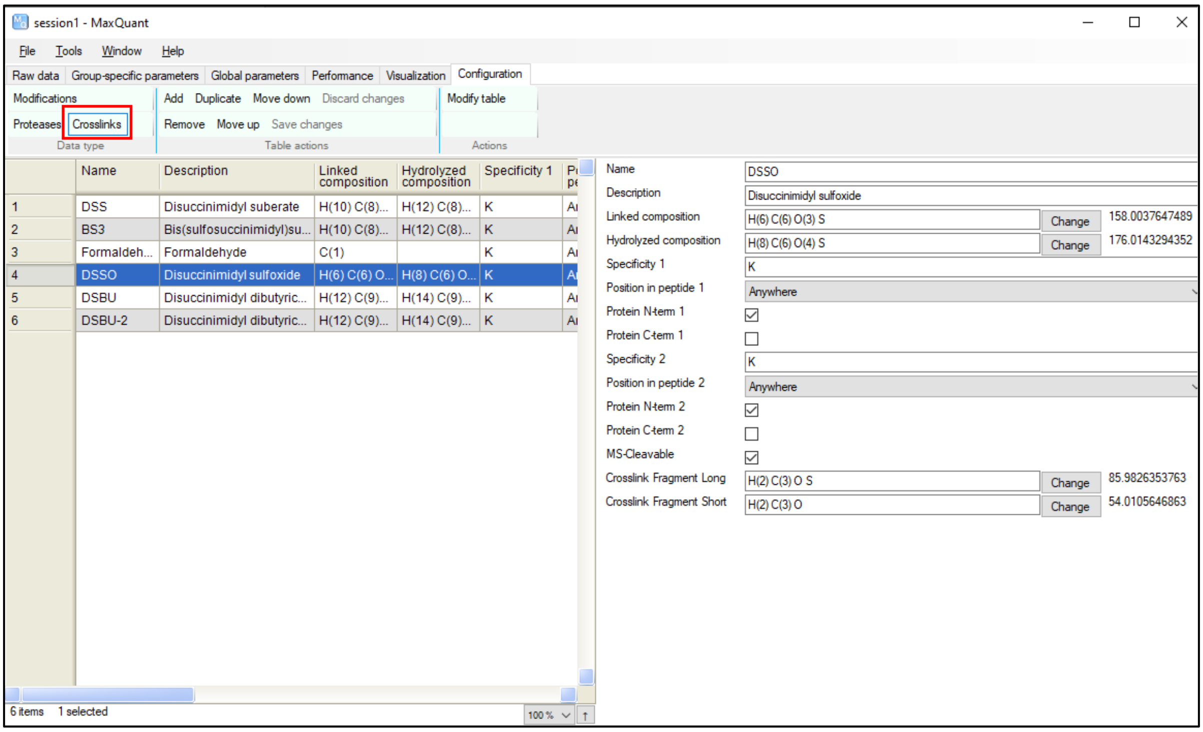

3. Increase the maximum number of missed cleavages and choose the enzyme of your choice.

In cross-linked peptide analysis, we tend to change this value to typically three, while the default MaxQuant value is 2 for the maximum missed cleavages

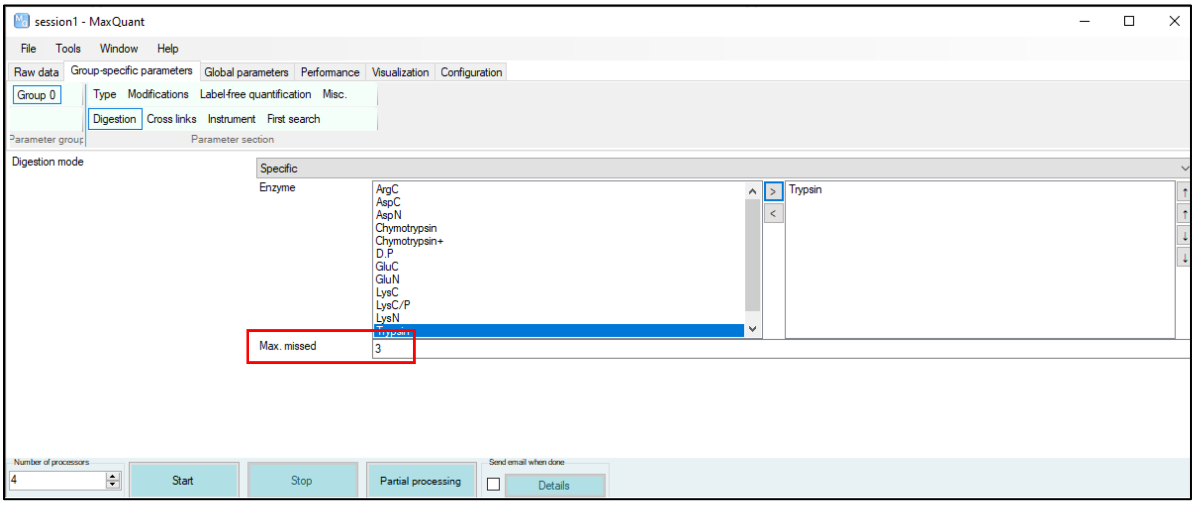

4. Turn on Peak refinement.

This new option can be enabled on *Misc.* tab under the *Group-specific parameters* tab.

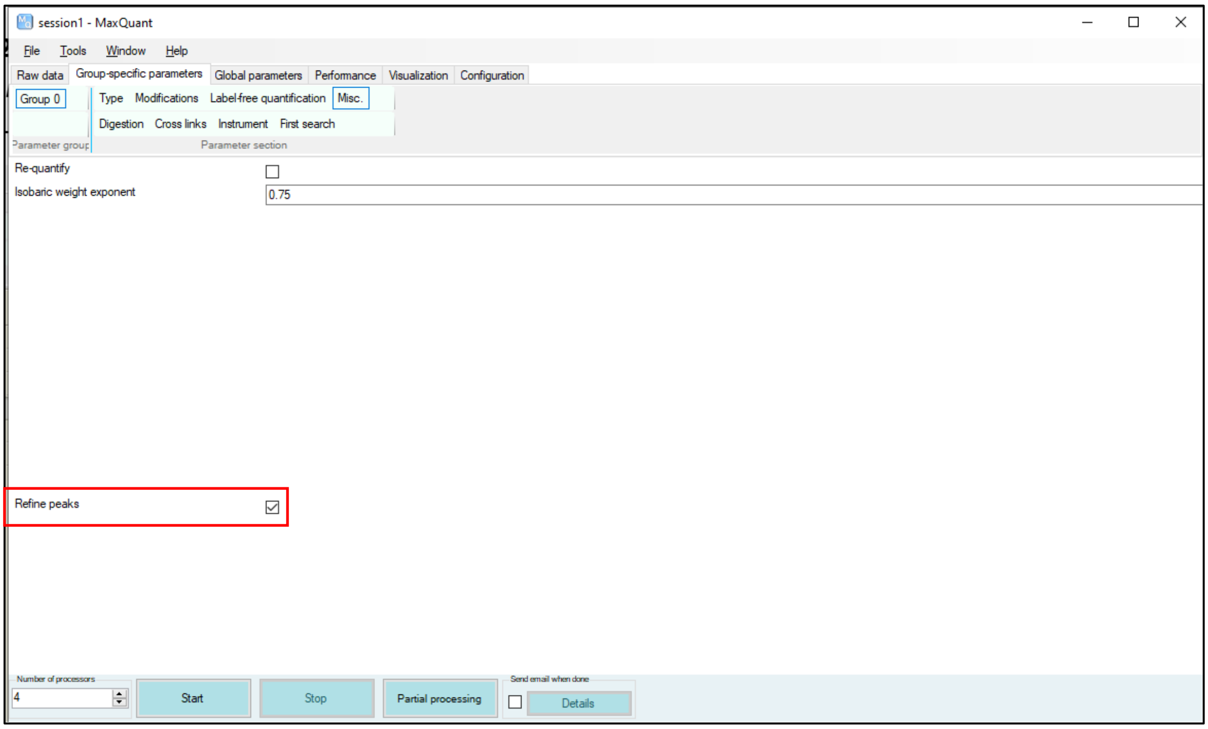

5. Set up protein sequence related information.

Add your fasta file but disable including contaminants. Decrease the peptide length to of your choice and increase the peptide mass to adapt cross-linked peptide searches (cross link software typically uses 6000 Da)

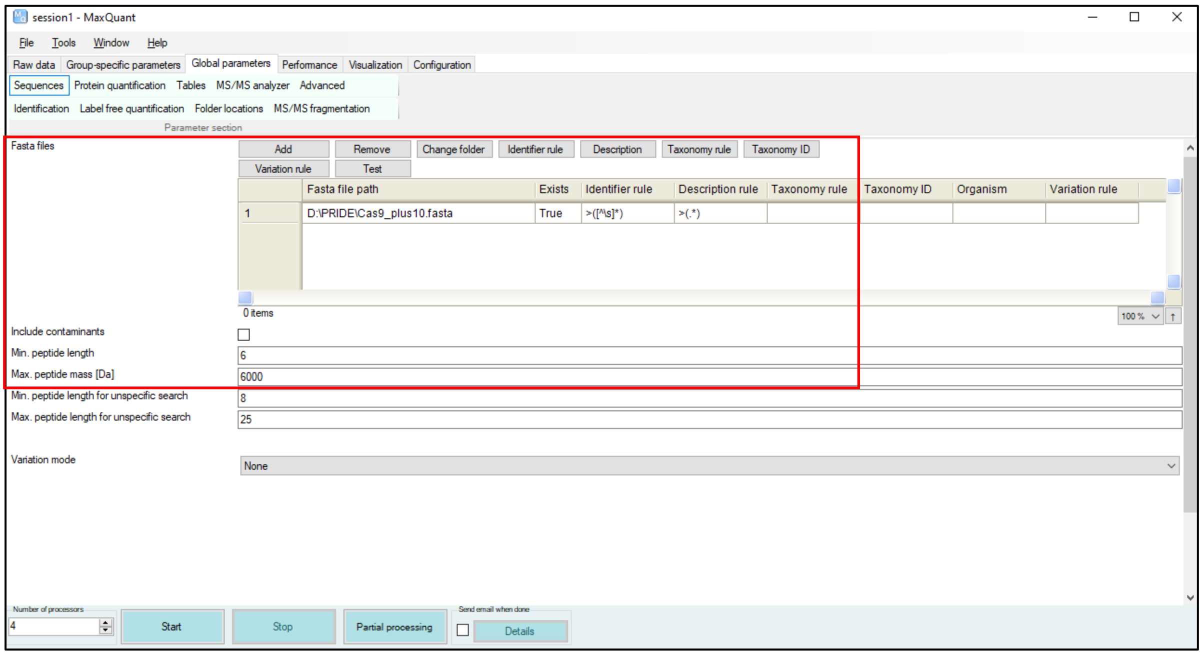

6. Set your FDR values.

Here you can currently disable “Second peptide” searches, this option is not functional for this current version.

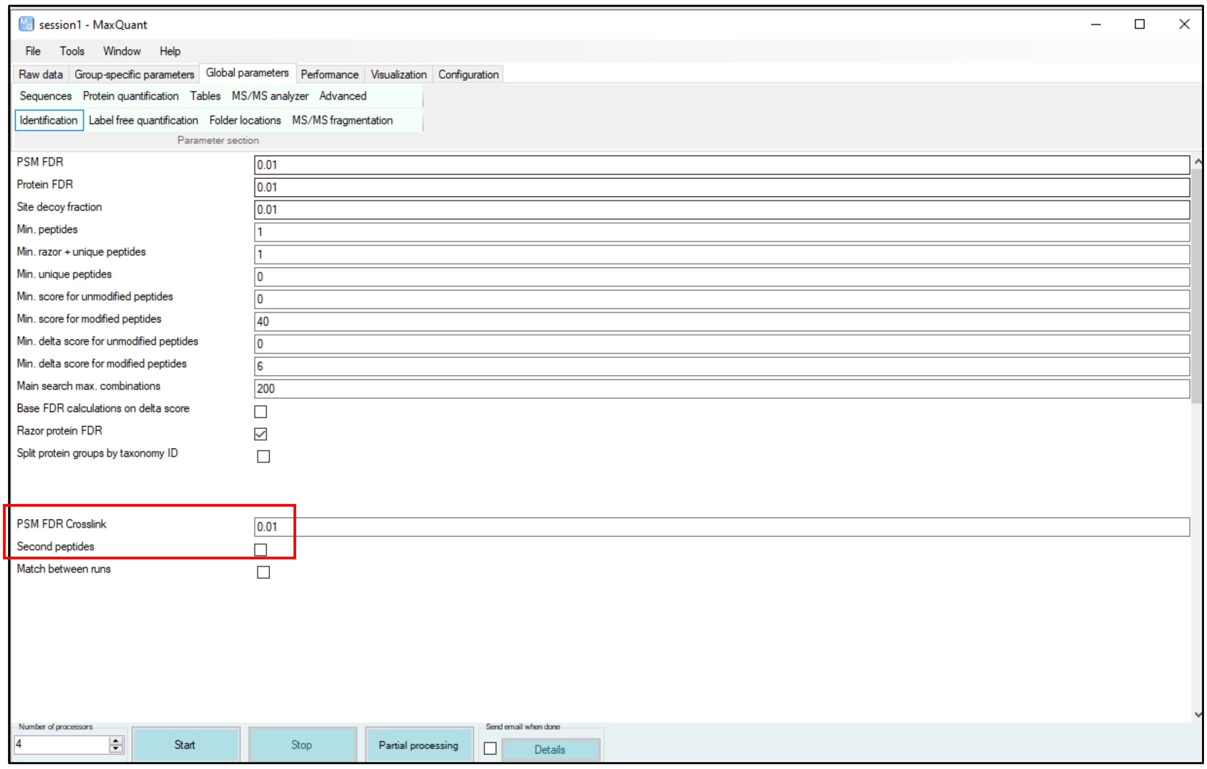

7. Disable losses and high charges for FTMS MS/MS analyser.

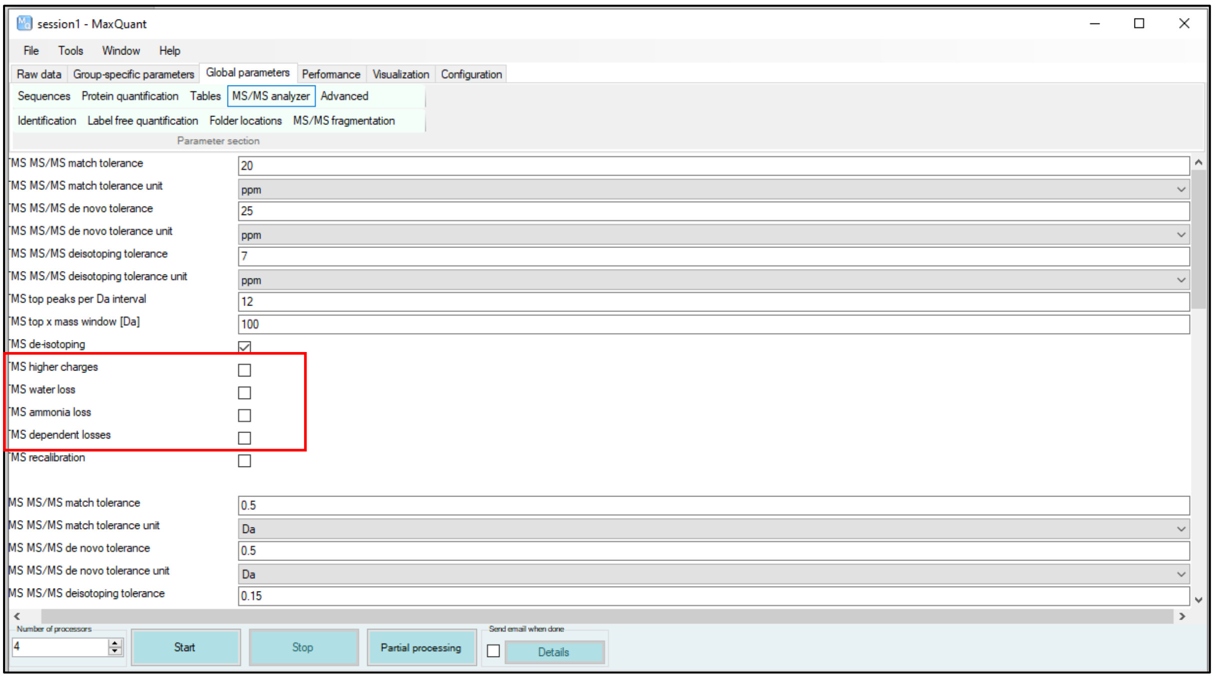

8. Note for Bruker TIMS instruments.

Increase the max charge from 4 to 6 because cross-linked peptides tend to have higher charge states.

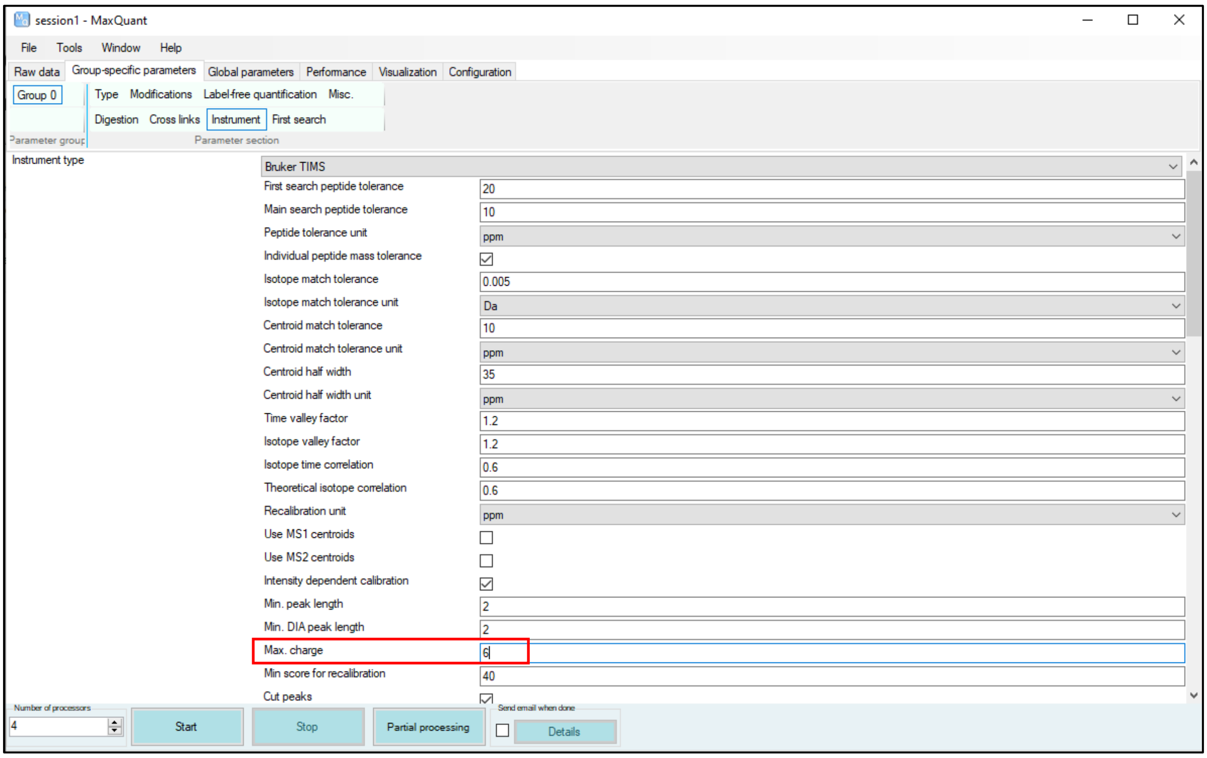

Note that if you previously analysed your TIMS TOF data set with earlier MaxQuant versions, you might encounter some problems. In this case, you should make a new mqpar.xml from the MaxQuant 2.0.4 version.

9. Inspect your results.

Go to Crosslink MS/MS table (where the information comes from the **crosslinkMsms.txt** table under the **combined/txt** folder after MaxQuant/MaxLynx analysis is finished). Select a row and make sure to be *MS/MS spectra* panel on the visualization. Then click on "*Display selected spectrum*" to view your identification. You can see the peptide sequence-based information on the *Peptide sequence* window under the *MS/MS spectra* panel.

We extended the fragment annotations here for cross link products.

- Any fragment coming from alpha or beta peptide has α or β, respectively.
- If a fragment contains an entire other peptide, then it has "Pep" (e.g. $y_4\alpha Pep$ is a y_4 fragment from an alpha peptide which is linked to a beta peptide).
- If it is MS-cleavable cross linking search, then it is possible to have some fragments with shorter and longer cross linker residual, then they are shown with "S" and "L", respectively (e.g. $y_4\alpha S$ is a y_4 fragment from an alpha peptide with a shorter cross linker residual).

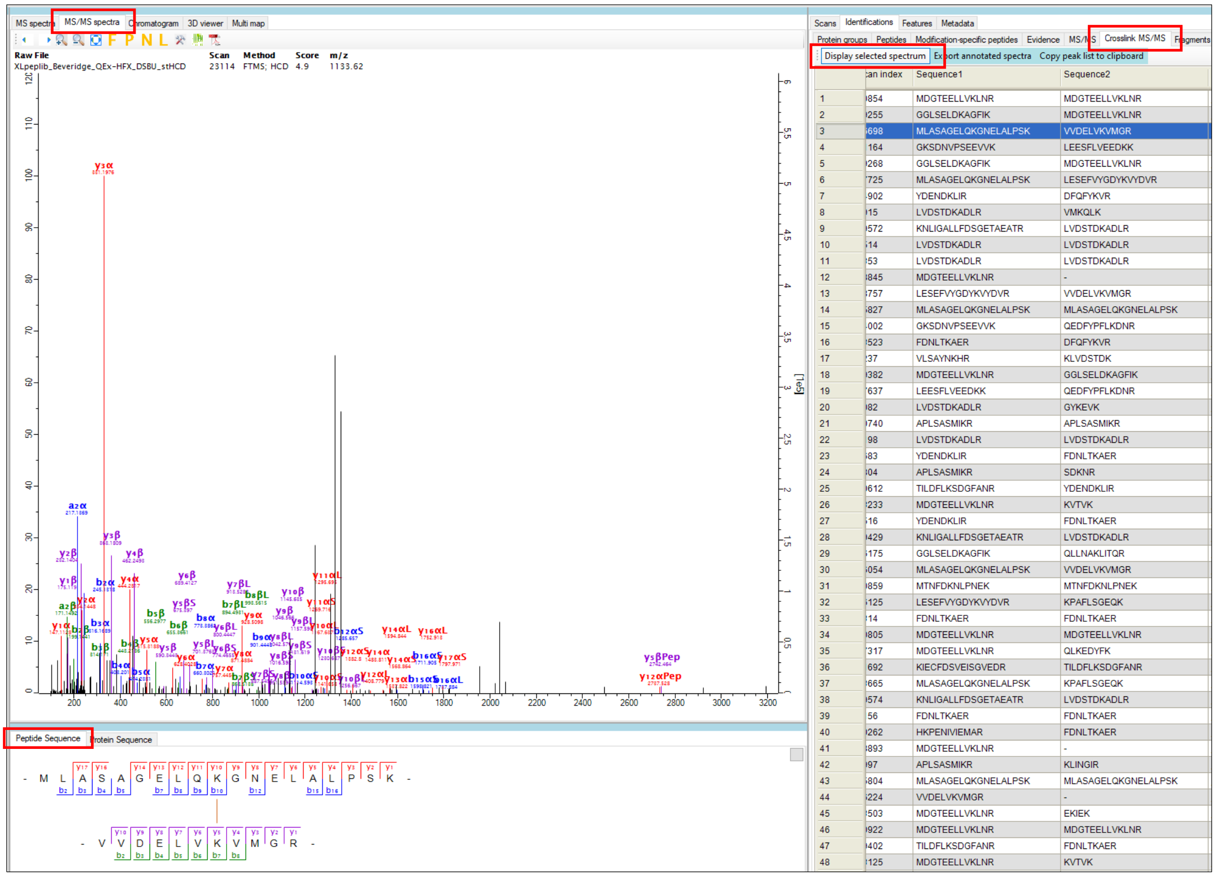

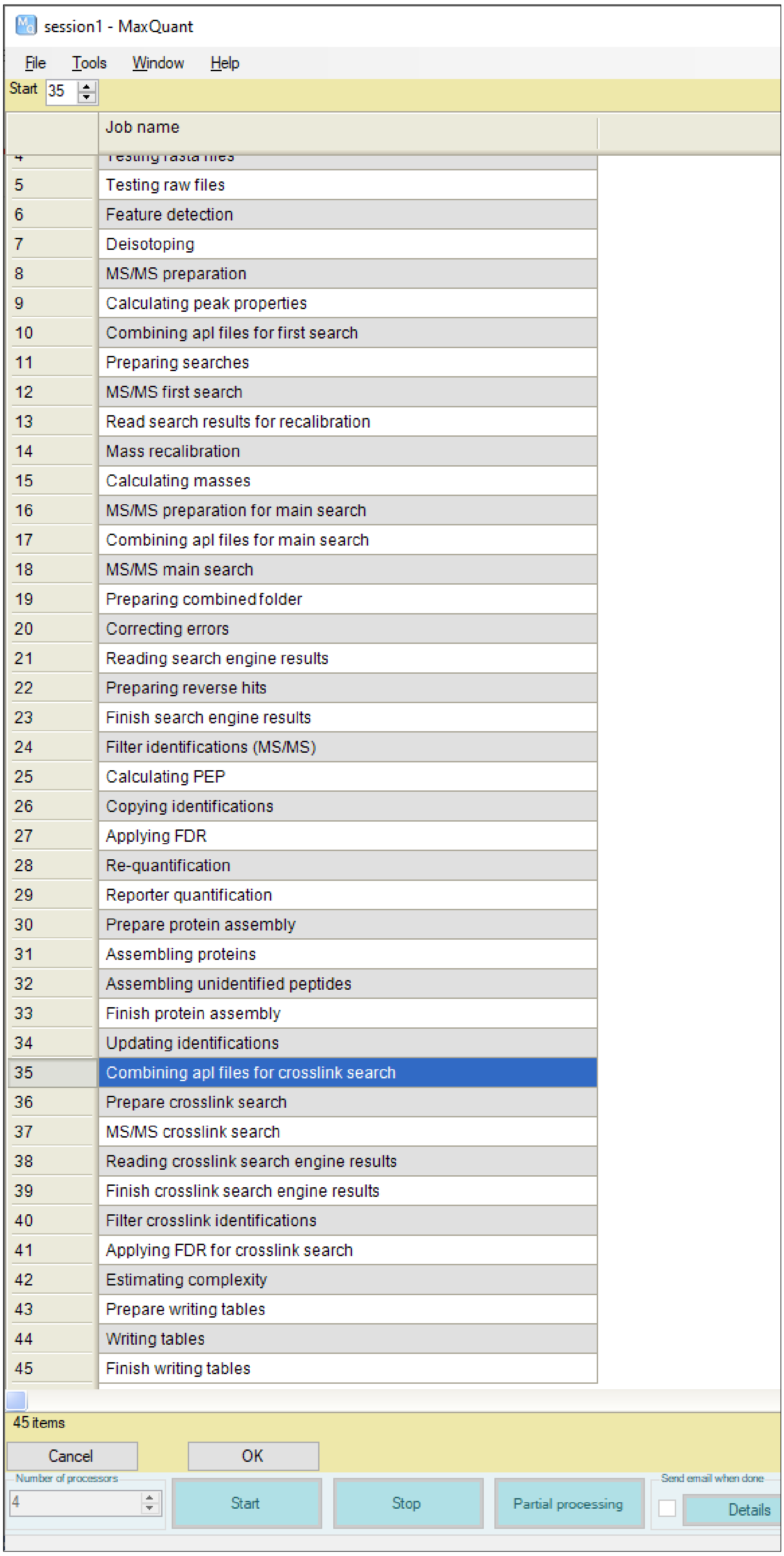

Supplementary Figure S1.

MaxLynx workflow for cross link search

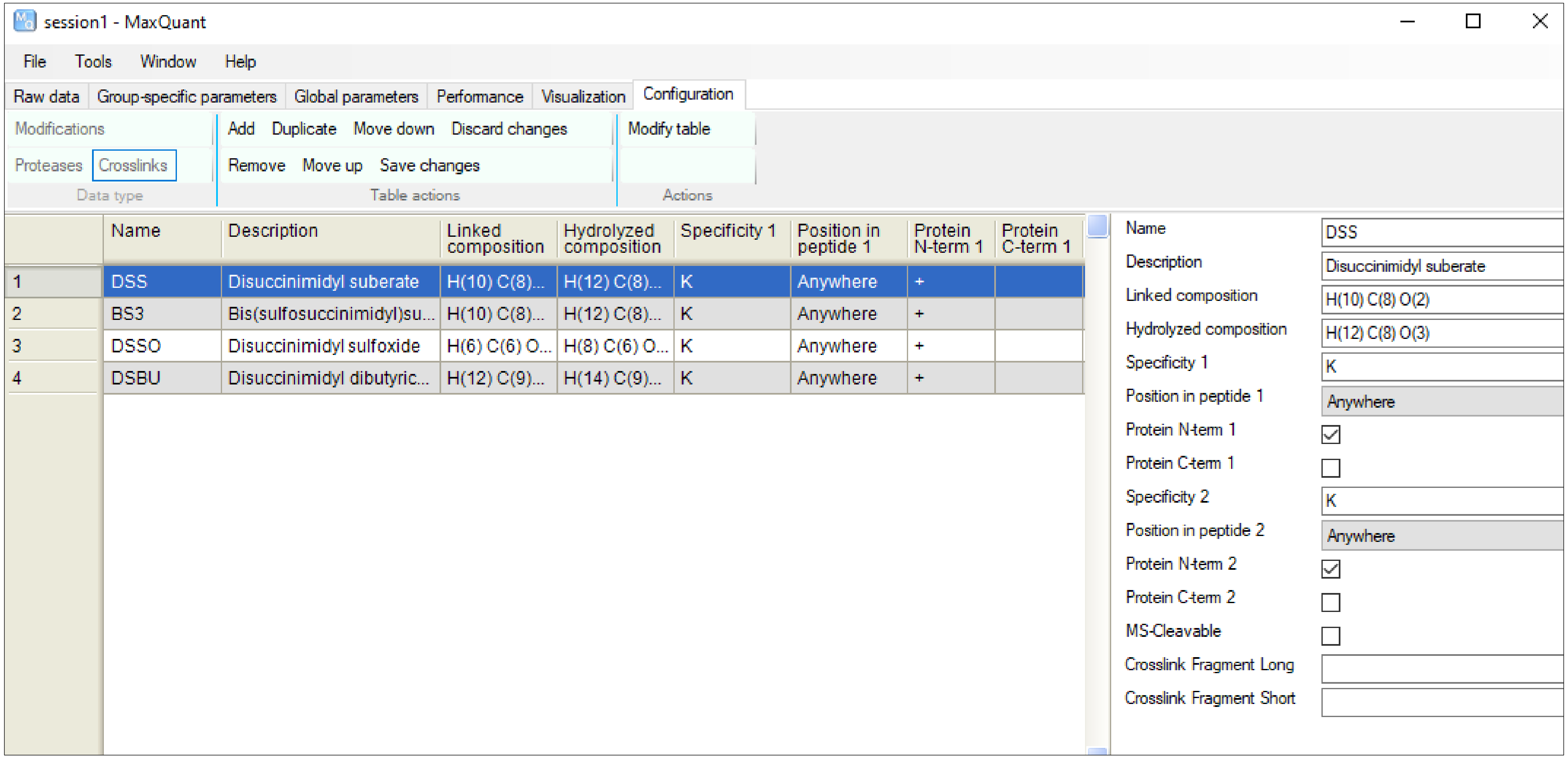

Supplementary Figure S2.

Crosslinks panel. User can easily configure a cross linker of interest through using the Crosslinks panel on the Configuration. See ***Step 2.c*** *on how to run MaxLynx guideline for the further details.*

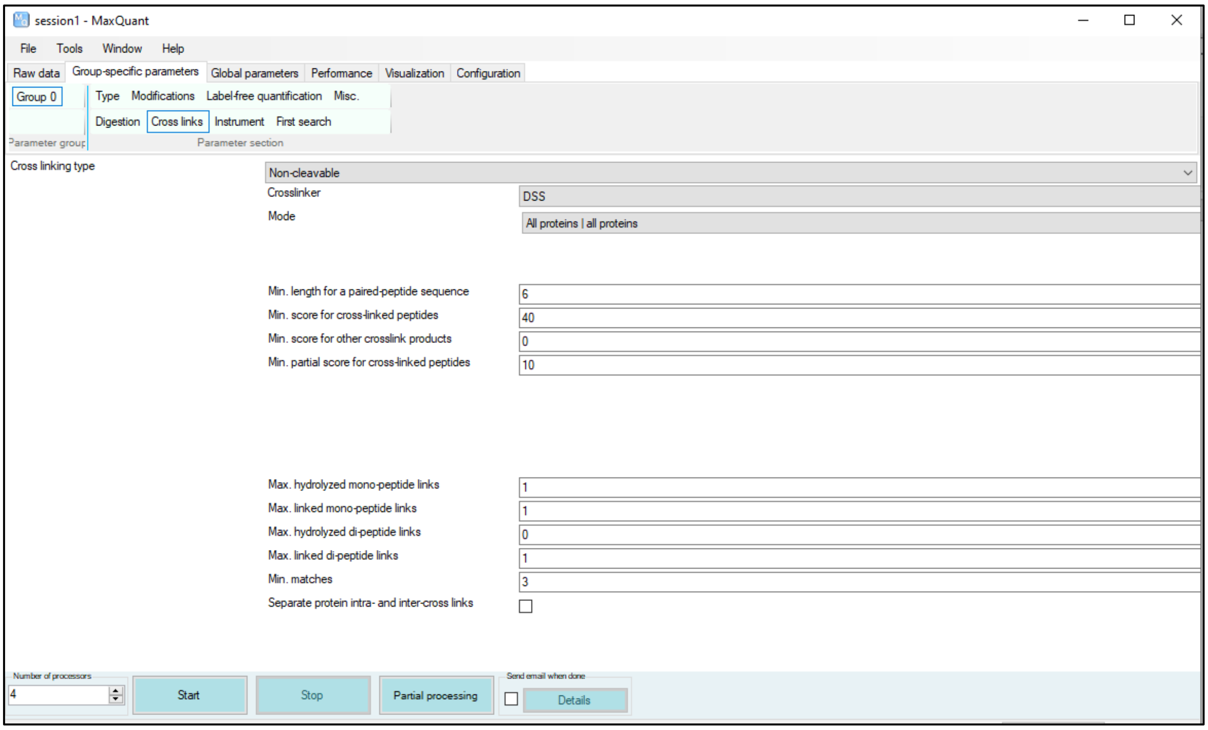

New group-specific parameter panel for cross link searches.

Supplementary Table S1.

The search settings used for the non-cleavable DSS data set. All the settings except for OpenPepXL and MaxLynx were taken from Beveridge and co-workers[1], whereas the OpenPepXL settings were from Netz and co-workers[2]. The MaxLynx settings were appended.

	MaxLynx (MaxQuant v2.0.4)	OpenPepXL (1.1)	pLink (2.3.5)	StavroX (3.6.0)	Xi (1.6.751)	Kojak (1.6.1)
**Cross link mass/ Da**	138.068	138.068	138.068	138.068	138.068	138.068
**Mono link mass/ Da**	156.079	156.079	156.079	156.079	156.079	156.079
**Cross linker reactivity**	K-K	K-K	K-K	K-K	K-K	K-K
**Fixed modification**	Carbamido-methyl [C]	Carbamido-methyl [C]	Carbamido-methyl [C]	Carbamido-methyl [C]	Carbamido-methyl [C]	Carbamido-methyl [C]
**Variable modification**	Oxidation [M]	Oxidation [M]	Oxidation [M]	Oxidation [M]	Oxidation [M]	Oxidation [M]
**Enzyme**	Trypsin	Trypsin	Trypsin	Trypsin	Trypsin	Trypsin
**Max. missed cleavages**	3	4	3	R:3 K:3	3	3
**Min peptide mass**	-	NA	500	500	-	500
**Max peptide mass**	6000	NA	6000	6000	-	6000
**Min peptide length**	5	5	5	5	5	-
**Max peptide length**	-	NA	60	-	-	-
**MS1 tolerance (ppm)**	5	6	5	5	5	5
**MS2 tolerance (ppm)**	20	20	20	20	20	Bin size 0.03 Thomson
**FDR calculation**	inbuilt (separate FDR)	TOPP tool XFDR	inbuilt	inbuilt	Xi FDR (1.1.27)	Percolator (3.02)
**FDR level**	PSM	PSM	PSM	PSM	PSM	PSM

Supplementary Table S2.

The search settings used for the MS-cleavable data sets. All the settings except for MaxLynx were taken from Beveridge and co-workers[1] and the MaxLynx settings were appended.

		**MaxLynx (MaxQuant v2.0.4)**	**XlinkX in proteome discoverer 2.3**	**MeroX 2.0 beta 5**
**DSBU**	**Cross link mass/ Da**	196.085	196.085	196.085
	**Bu-fragment/ Da**	85.053	-	85.053
	**BuUr-fragment/ Da**	111.032	-	111.032
**DSSO**	**Cross link mass/ Da**	158.004	158.004	158.004
	**Alkene/ Da**	54.011	-	54.011 (essential)
	**Thiol/ Da**	85.983	-	85.983 (essential)
	**Sulfenic acid/ Da**	-	-	103.993
**Cross linker reactivity**		K-K	K-K	K-K
**Fixed modification**		Carbamidomethyl [C]	Carbamidomethyl [C]	Carbamidomethyl [C]
**Variable modification**		Oxidation [M]	Oxidation [M]	Oxidation [M]
**Enzyme**		Trypsin	Trypsin	Trypsin
**Max. missed cleavages**		3	3	R:3 K:3
**Min peptide mass**		-	500	500
**Max peptide mass**		6000	6000	6000
**Min peptide length**		5	5	5
**MS1 tolerance (ppm)**		5	5	5
**MS2 tolerance (ppm)**		20	20	20
**S/N ratio**		-	1.5	1.5
**FDR calculation**		inbuilt (separate FDR)	inbuilt	inbuilt
**FDR level**		PSM	PSM	PSM

Supplementary Table S3.

Number of cross linked peptides to spectrum matches (CSMs) in the DSS data set at **1% FDR**. The OpenPepXL results were taken from Netz and co-workers [2] whereas the results of pLink, StavroX and Xi from Beveridge and co-workers [1].

**Number of cross linked peptides to spectrum matches at FDR=1%**						
	**Correct**			**Incorrect**		
**Search engine**	**R1**	**R2**	**R3**	**R1**	**R2**	**R3**
**MaxLynx**	737	940	880	6	18	11
**OpenPepXL**	368	506	365	4	5	4
**pLink**	594	644	585	10	13	25
**StavroX**	265	157	160	4	0	1
**Xi**	312	352	438	2	4	5

Supplementary Table S4.

Number of unique cross links in the DSS data set at **1% FDR**. The OpenPepXL results were taken from Netz and co-workers [2] whereas the results of pLink, StavroX and Xi from Beveridge and co-workers [1].

**Number of cross links at FDR=1%**						
	**Correct**			**Incorrect**		
**Search engine**	**R1**	**R2**	**R3**	**R1**	**R2**	**R3**
**MaxLynx**	227	240	223	6	14	9
**OpenPepXL**	161	196	148	2	4	3
**pLink**	215	218	189	9	12	22
**StavroX**	124	91	90	4	0	1
**Xi**	141	152	163	2	3	5

Supplementary Table S5.

The number of cross links identified on the MS-cleavable cross linker data sets, DSBU and DSSO respectively at **1% FDR**. MeroX and XlinkX results were taken from the Beveridge and co-workers[1].

**Number of cross links at FDR 1%**		
**Cross linker, Search engines**	**Correct**	**Incorrect**
**DSBU, MaxLynx**	242	10
**DSBU, MeroX, Rise**	207	11
**DSBU, MeroX, Riseup**	237	15
**DSBU, XlinkX**	120	37
**DSSO, MaxLynx**	185	3
**DSSO, MeroX, Rise**	124	1
**DSSO, MeroX, Riseup**	149	19
**DSSO, XlinkX**	128	53

MaxLynx-specific parameter analysis:

a. Combination with total-score and partial score values

We have performed an analysis for parameter scanning and we compared the MaxLynx results (MaxQuant v.2.0.3) with the different setting combinations of total score (the cross linked peptide score) as 0, 20 and 40 and the partial-score as 0, 10 and 20. Note that the default min-match parameter was left as 3. Here, we did not separate protein intra- and inter-cross links. We used the default MS/MS analyzer setting. We performed this analysis for DSS, DSBU, DSSO data sets by Beveridge *et al*.

DSS data set by Beveridge et al

As seen on the left of the **Supplementary Figure S4** when total-score was set to 0, the distributions for the correct CSMs shift down as corresponding to increasing the partial-score. This can be expected because increased partial-score will also remove some correct CSMs with very low-total-scores. The highest number of incorrect CSMs was found here when no score-filtering applied (total-score=0 and partial-score=0). When partial-score was set to 10, the number of incorrect CSMs decreases.

When total-score was set to 40 (**the Supplementary Figure S4** on the right), the effect of the partial score almost disappears on the number of correct CSMs and the correct CSMs distributions, partial-scores=0, 10 and 20, became almost the same for the number of the correct CSMs. This change was not observed for the distributions for the incorrect CSMs and the distribution for the incorrect CSMs across the three partial-score remains more less the same.

The results for total-score=20 (**the Supplementary Figure S4** on the middle), were intermediate and it was observed that slightly less correct CSMs compared to the results with total-score=0. The number of incorrect CSMs were remained the same for the partial-score=10 with the number from the total-score=0 and partial-score=10.

The **Supplementary Figure S5** shows the results from the same analysis but for the number of unique crosslinks. The almost same patterns observed in Figure 1 can be also seen here.

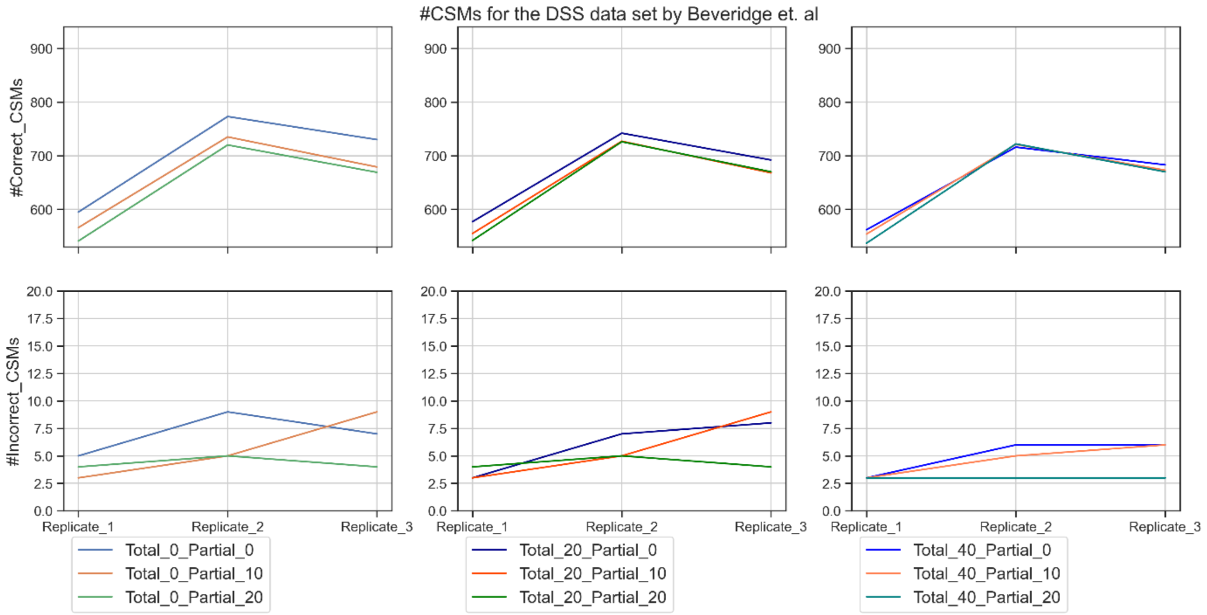

Supplementary Figure S4.

Number of CSMs for the DSS data set by Beveridge et al. Each plot contains the results from the three replicates from the data set, which shown as Replicate_1, Replicate_2 and Replicate_3. The plots on the upper panel show the distributions for the number of correct CSMs whereas the plots on the lower panel show the distributions for the number of incorrect CSMs. Two plots on the left shows the results from total-score=0 with combination of partial-scores from 0, 10 and 20. The middle plots for total-score=20 and the right plots for total-score=40 are shown with combination of partial-scores from 0, 10 and 20.

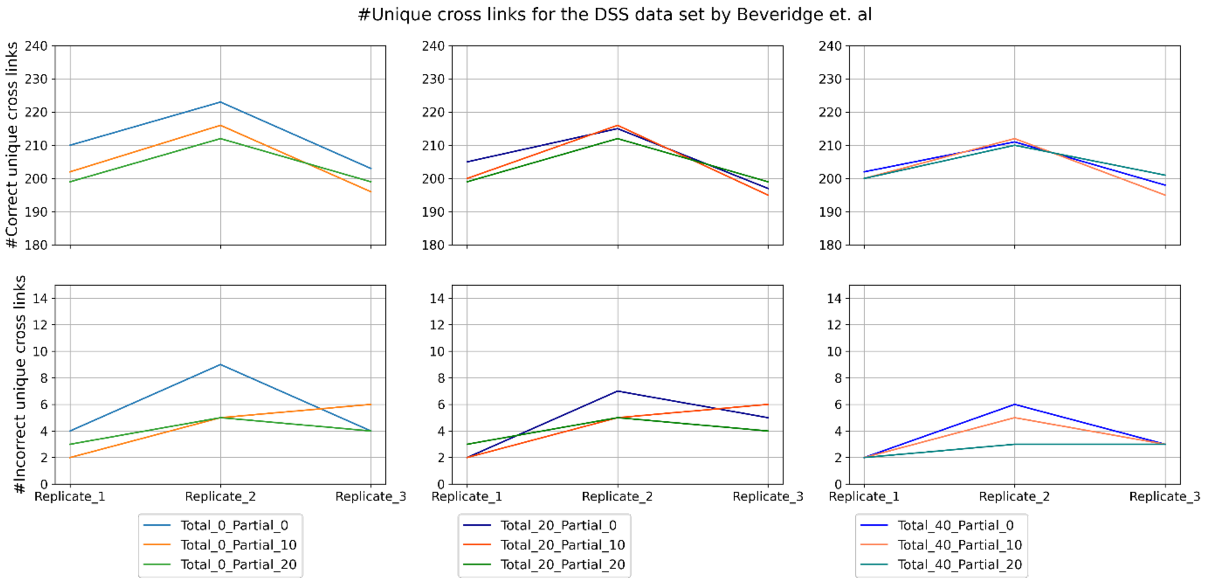

Supplementary Figure S5.

Number of unique cross links for the DSS data set by Beveridge et al. Each plot contains the results from the three replicates from the data set, which shown as Replicate_1, Replicate_2 and Replicate_3. The plots on the upper panel show the distributions for the number of correct cross links whereas the plots on the lower panel show the distributions for the number of incorrect cross links. Two plots on the left shows the results from total-score=0 with combination of partial-scores from 0, 10 and 20. The middle plots for total-score=20 and the right plots for total-score=40 are shown with combination of partial-scores from 0, 10 and 20.

DSBU data set by Beveridge et al

As seen on the **Supplementary Figure S6**, there is a trend on when a partial-score increases, the number of correct CSMs decreases for the DSBU data set

At fixed total score (total-score=0, total-score=20 or total-score=40), partial-score=10 keeps a similar number of correct CSMs but removes more than one-third of incorrect CSMs compared the results at the partial-score=0. Increasing the partial-score=20 resulted in more decreasing number of correct CSMs, so the decrease in the incorrect CSMs was not compensated.

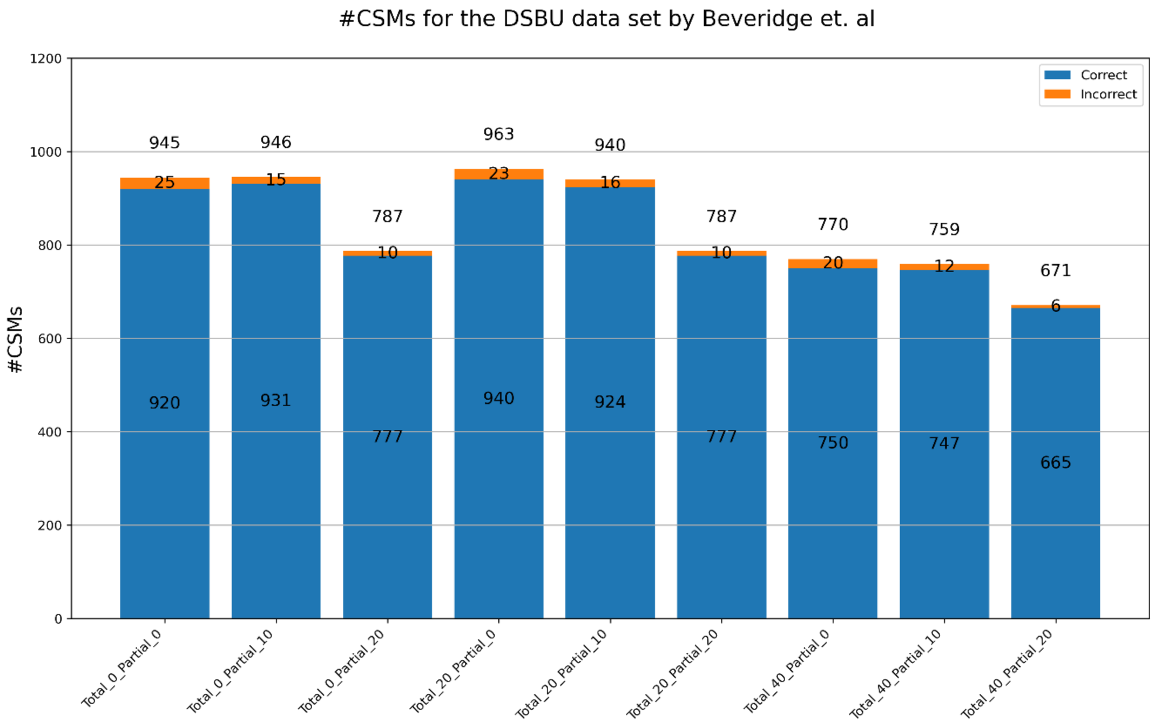

Supplementary Figure S6.

Number of CSMs at the DSBU data set by Beveridge et al. The used settings, the combination of the total-score and partial-score, were noted on the x-axis. The correct CSMs are shown in blue whereas the incorrect ones are in orange. The number of correct CSMs and incorrect CSMs were shown in the center of the corresponding bars and the sum of the correct and incorrect CSMs are displayed on top of each bar.

We also compared the number of unique cross links at the DSBU data set (**Supplementary Figure S7**). Although the effect of increasing both total-score and partial-score was not as strong as the results for CSMs, this trend is still similar to the **Supplementary Figure S6.** We observed that increasing the partial-score from 0 to 10 reduced the number of incorrect cross links but keep more less the same number of correct cross links.

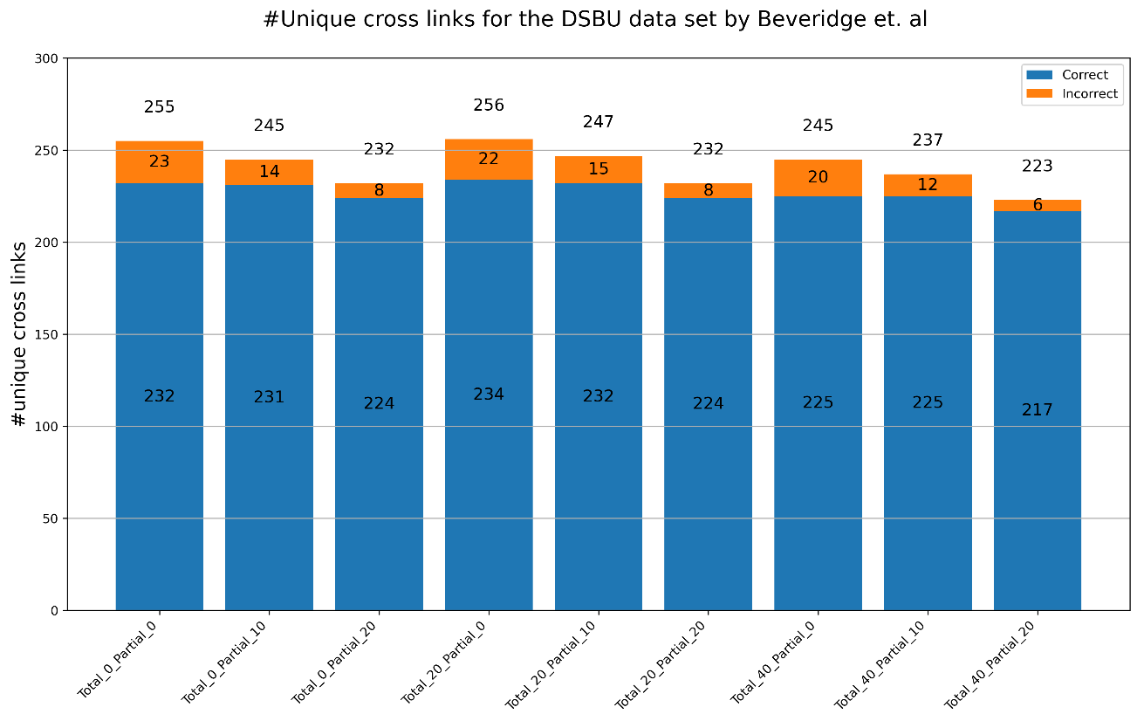

Supplementary Figure S7.

Number of unique cross links at the DSBU data set by Beveridge et al. The used settings, the combination of the total-score and partial-score, were noted on the x-axis. The correct unique cross links are shown in blue whereas the incorrect ones are in orange. The number of correct and incorrect unique cross links were shown in the center of the corresponding bars and the sum of the correct and incorrect ones are displayed on top of each bar.

DSSO data sets by Beveridge et al

The CSM results are at first surprising because there is no clear pattern compared to the other results (**Supplementary Figure S8**). When we have a close look at total-score=0 and partial-score=0, we observed that there are high-scored decoys affecting the results. The affect can be also due to the lower number of CSMs for this data set and any high-scored decoy CSMs have more profound affect. Unlike typical proteomics search, XL-MS have some decoys that are composed of target and decoy protein. Most of these high-scored decoys are half-decoy (decoy-is-linked-to-target). We expect that separate FDR could help here. With high-score filtering, total-score=40, this obscure result is diminished.

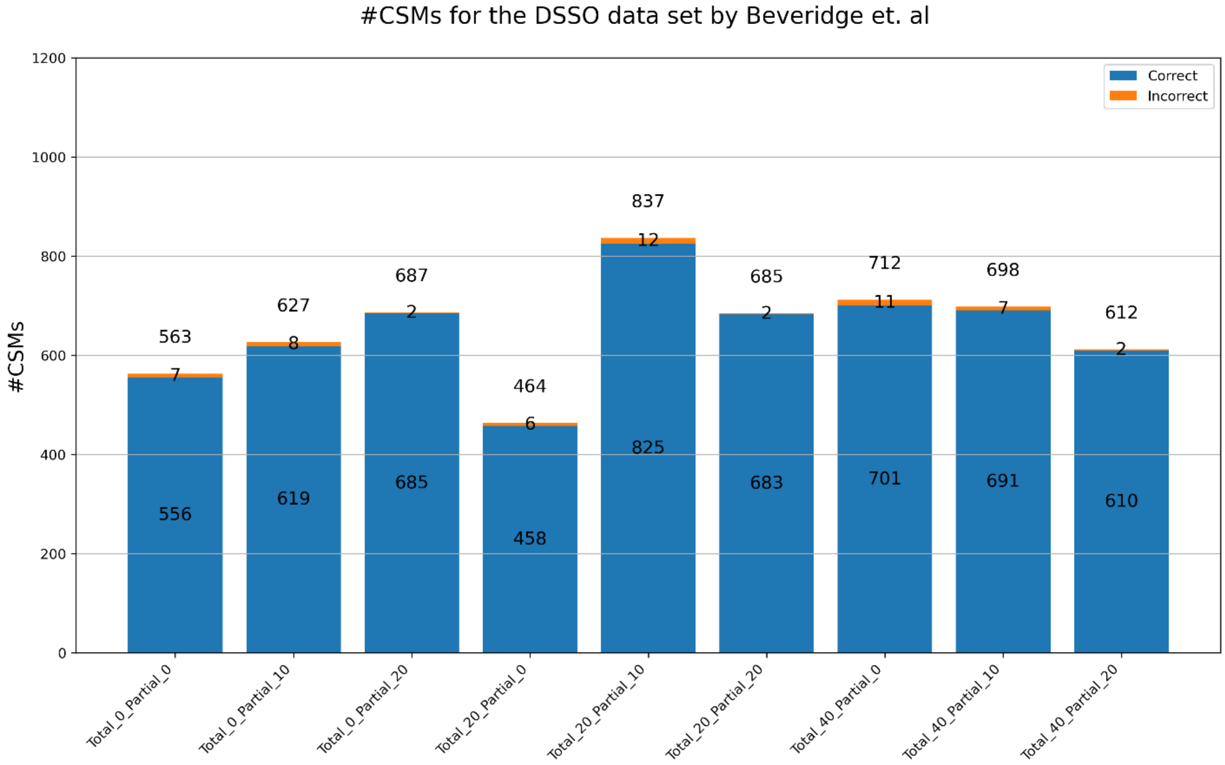

Supplementary Figure S8.
Number of CSMs at the DSSO data set by Beveridge et al. The used settings, the combination of the total-score and partial-score, were noted on the x-axis. The correct CSMs are shown in blue whereas the incorrect ones are in orange. The number of correct CSMs and incorrect CSMs were shown in the center of the corresponding bars and the sum of the correct and incorrect CSMs are displayed on top of each bar.

The distribution of unique cross links is similar to what we observed for the trend on the CSMs for the DSSO data set. This is not surprising because CSMs are cross links are correlated (**Supplementary Figure S9**).

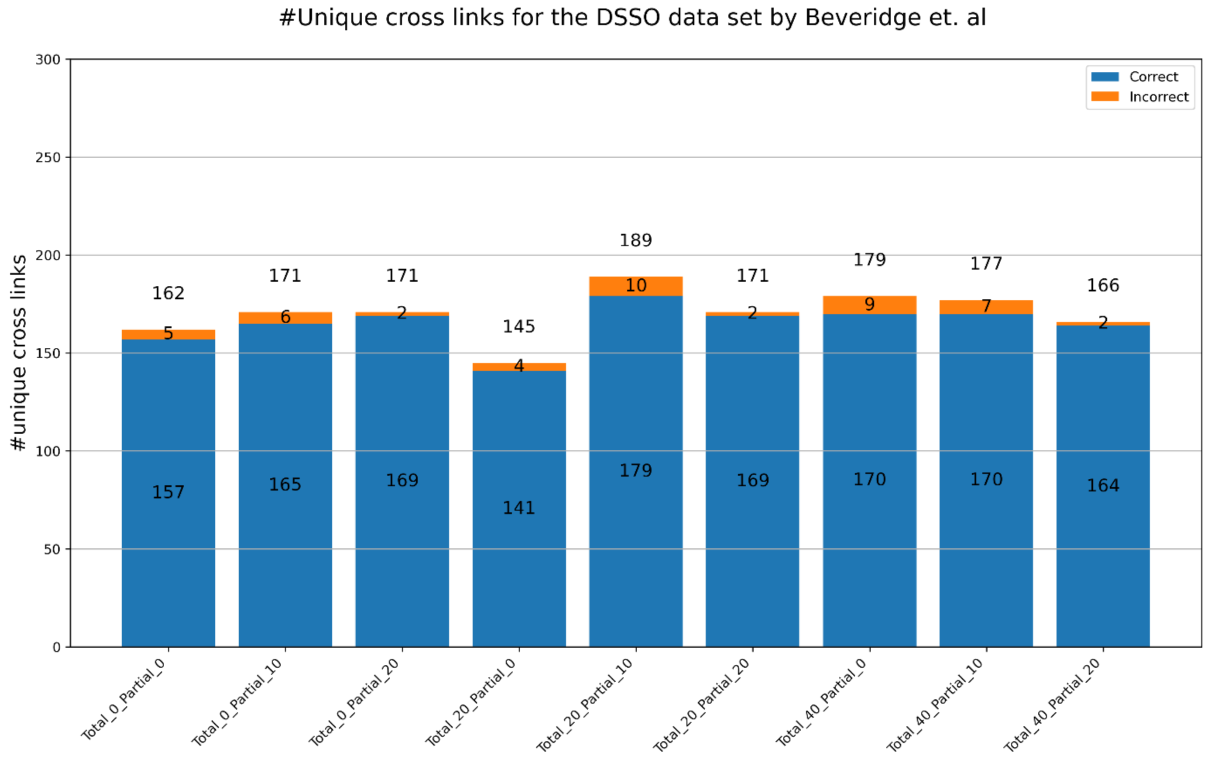

Supplementary Figure S9.

Number of unique cross links at the DSSO data set by Beveridge et al. The used settings, the combination of the total-score and partial-score, were noted on the x-axis. The correct unique cross links are shown in blue whereas the incorrect ones are in orange. The number of correct and incorrect unique cross links were shown in the center of the corresponding bars and the sum of the correct and incorrect ones are displayed on top of each bar.

In general, we did not observe a clear pattern for the total-score. Therefore, we suggest to leave this value as 0. However, we observed a consistent behavior on the partial-score value to help reducing incorrect hits, with the optimum value as 10, therefore we recommend this using the filter option based on the partial-score.

b. Effect on separating protein intra- and inter-crosslinks for FDR control

We have performed several more MaxLynx runs to evaluate the effect of using separate FDR together with the combination of total-score and partial-score. Note that we kept the MS/MS analyzer settings as default to see its direct affect for the synthetic data set by Beveridge *et al*. In addition to the synthetic data set, we did six different MaxLynx runs for the proteome-wide data set (total score=0; partial score=0, 10 or 20; separate FDR=on/off).

DSS data set by Beveridge et al

We observed that there was a slight increase in correct CSMs compared to without separating CSMs into inter- and intra-protein cross links at total-score=0 and partial-score=10 but the other settings did not show a clear difference on the number of correct CSMs (sometime less, sometimes more CSMs) (**Supplementary Figure S10**). The used database contains one Cas9 protein and 10 contaminant proteins (such as keratin). Note that this database was not created by us, but we used the provided database by Beveridge *et al (CITE).* At the total-score=0 and partial-score=10, the correct CSMs were 566, 735, 679 and the incorrect CSMs were 3, 5 and 9. Here there was only one CSM from inter-protein cross link, which was between two different contaminant protein. The same settings but with separating FDR, the correct CSMs were increased to 571, 742, 684 and the incorrect CSMs were 3, 11 and 10 (4 of these incorrect CSMs were related to contaminants). In most of the settings, there was a slight increase in the number of the incorrect CSMs. This is related to also considering these possible cross links involving contaminants.

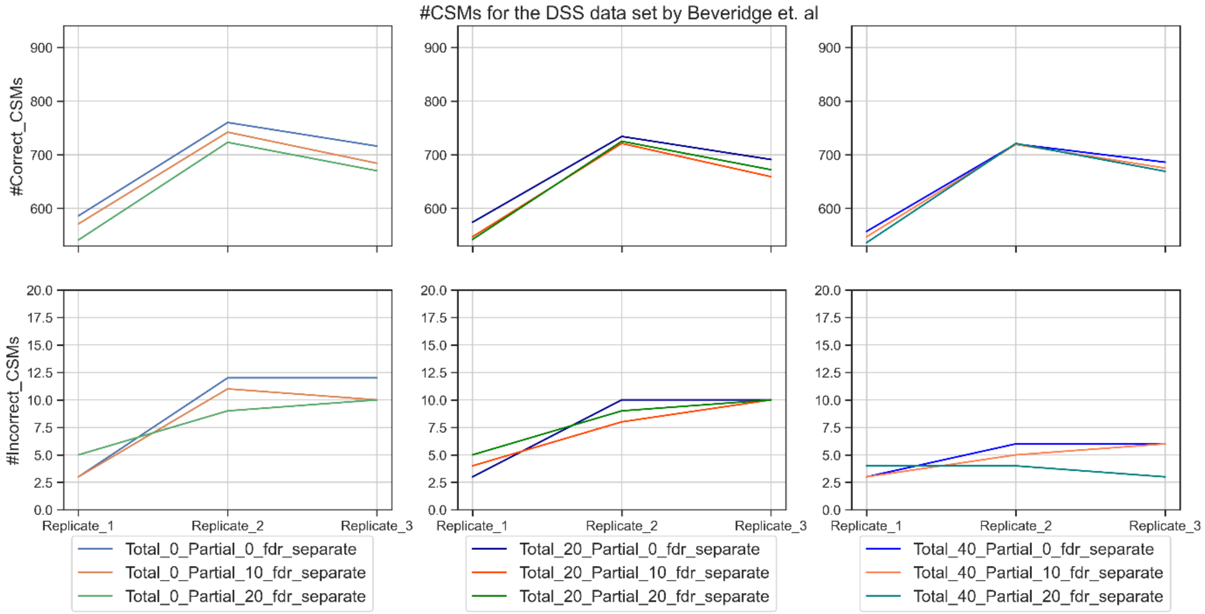

Supplementary Figure S10.

Number of CSMs for the DSS data set by Beveridge et al, when protein inter- and intra-cross links were split. Each plot contains the results from the three replicates from the data set, which shown as Replicate_1, Replicate_2 and Replicate_3. The plots on the upper panel show the distributions for the number of correct CSMs whereas the plots on the lower panel show the distributions for the number of incorrect CSMs. Two plots on the left shows the results from total-score=0 with combination of partial-scores from 0, 10 and 20. The middle plots for total-score=20 and the right plots for total-score=40 are shown with combination of partial-scores from 0, 10 and 20.

The effect of separating protein cross links for the FDR calculation is less obvious for the unique cross links compared to the change on the CSMs. Moreover, the distribution for unique cross links corresponds well to the changes to CSMs (**Supplementary Figure 11**).

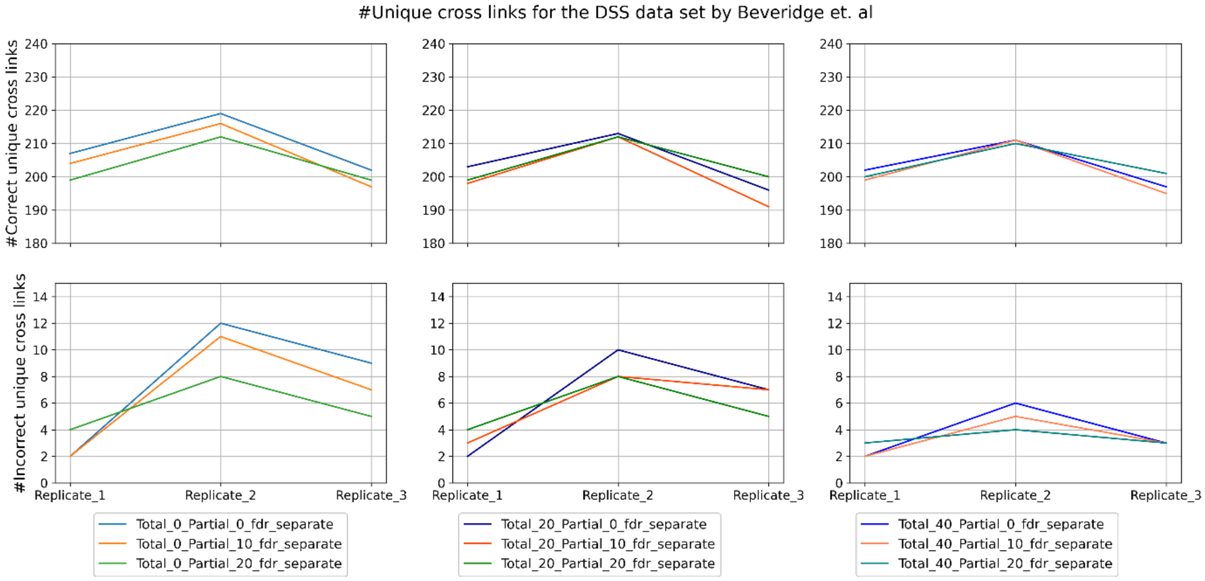

Supplementary Figure S11.

Number of unique cross links for the DSS data set by Beveridge et al, when protein inter- and intra-cross links were split. Each plot contains the results from the three replicates from the data set, which shown as Replicate_1, Replicate_2 and Replicate_3. The plots on the upper panel show the distributions for the number of correct cross links whereas the plots on the lower panel show the distributions for the number of incorrect cross links. Two plots on the left shows the results from total-score=0 with combination of partial-scores from 0, 10 and 20. The middle plots for total-score=20 and the right plots for total-score=40 are shown with combination of partial-scores from 0, 10 and 20.

DSBU and DSSO data sets by Beveridge et al

For both of the MS-cleavable data sets, the number of correct CSMs were increased clearly. The number of incorrect CSMs did not typically increased but rather stayed the same or similar value.

The effect of separating FDR only increased the number of correct unique cross links at total-score=0 settings, when this was increased to higher number there were mostly no changes.

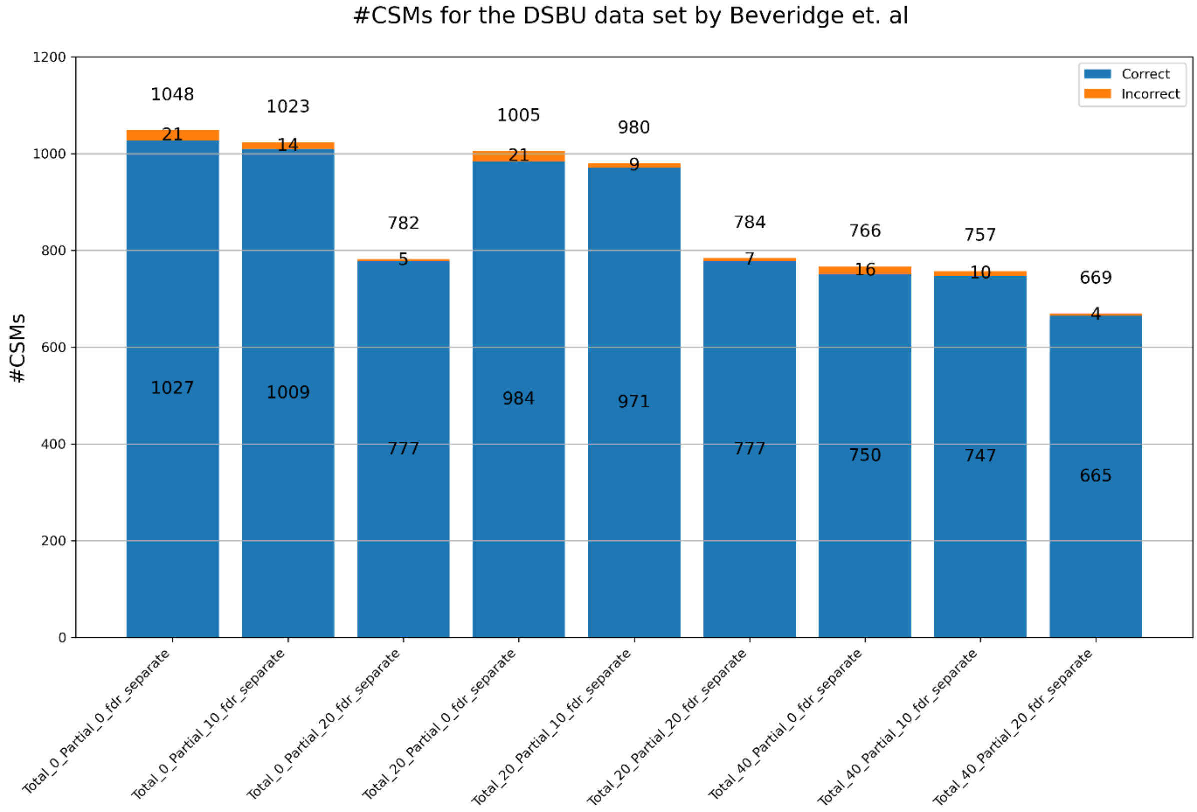

Supplementary Figure S12.

Proteome-wide data analysis

We have also analyzed the effect of separate protein intra- and inter-cross links FDR on the proteome wide study, the proteome-wide dataset of cross-linked D. melanogaster cell lysate (PXD012546). The effect of separating FDR has more profound on MS-cleavable cross linker on such data set. Because, especially with MS-cleavable cross linkers, we find very few intra-decoy-proteins unless, observing so many inter-decoy-proteins (**Supplementary Figure 6**).

Supplementary Table S6.

Supplementary Table S6. Results for different score and partial score settings together with separate FDR option at proteome-wide study

Score	Partial	FDR type	#CSMs	#unique cross-links	#unique intra-molecule cross-links	#unique inter-molecule cross-links
0	0	Separate FDR=OFF	29169	7161	5942	1219
0	0	Separate FDR=ON	49352	9250	8844	406
0	10	Separate FDR=OFF	31091	7327	6085	1242
0	10	Separate FDR=ON	48019	9035	8662	373
0	20	Separate FDR=OFF	35735	7842	6516	1326
0	20	Separate FDR=ON	42685	8302	7991	311

c. Effect of high-charged theoretical peaks and neutral losses

We observed that excluding high-charged and neutral loss related peaks, the identification rates were increased for FTMS analyzers (**Table S7- S12**). We have also run with excluding only neutral losses but the combination together with excluding high charge, we observed the highest affect. Including much more peaks on theoretical spectrum for a cross linked peptide over populated and increased the chance to make a match by mistake. Furthermore, We have tested also on TIMS-TOF data set but we observed that this leads typically to increase in number of false identifications, in addition to increase in overall identification numbers. This could be due to TOF analyzers are not as accurate as FTMS analyzers. Therefore, for cross linking example for FTMS analyzers, we recommend to excluding these default MS/MS analyzer settings for FTMS analyzers but not TIMS-TOF **(Table S13-S14).**

Supplementary Table S7.

Table S6. Number of correct and incorrect CSMs for the DSS data set by Beveridge *et al* for different score and partial score values together with specific change (either none "-"; calculating FDR on separating protein inter- and intra-crosslink (fdr_separate); excluding neutral losses peak additions (NO_loss); excluding both neutral losses and high-charged peaks (NO_loss_NO_highcharge)<br>Result are at FDR=1%								
**Score**	**Partial**	**Additional change**	**#Correct_1**	**#Correct_2**	**#Correct_3**	**#Incorrect_1**	**#Incorrect_2**	**#Incorrect_3**
0	0	-	595	773	730	5	9	7
0	0	fdr_separate	586	760	716	3	12	12
0	0	NO_loss	724	902	855	5	15	12
0	0	NO_loss_NO_highcharge	738	929	871	3	13	7
0	10	-	566	735	679	3	5	9
0	10	fdr_separate	571	742	684	3	11	10
0	10	NO_loss	711	889	838	3	8	12
0	10	NO_loss_NO_highcharge	726	922	866	3	13	6
0	20	-	541	720	669	4	5	4
0	20	fdr_separate	541	723	670	5	9	10
0	20	NO_loss	624	796	751	2	4	5
0	20	NO_loss_NO_highcharge	657	831	786	3	5	3
20	0	-	577	742	692	3	7	8
20	0	fdr_separate	574	734	691	3	10	10
20	0	NO_loss	718	885	843	2	10	10
20	0	NO_loss_NO_highcharge	734	920	863	2	11	8
20	10	-	555	727	668	3	5	9
20	10	fdr_separate	547	721	659	4	8	10
20	10	NO_loss	706	878	829	2	7	9
20	10	NO_loss_NO_highcharge	725	917	858	2	10	9
20	20	-	542	726	670	4	5	4
20	20	fdr_separate	542	725	672	5	9	10
20	20	NO_loss	624	796	751	2	4	5
20	20	NO_loss_NO_highcharge	657	831	788	3	5	3
40	0	-	562	716	683	3	6	6
40	0	fdr_separate	557	720	686	3	6	6
40	0	NO_loss	653	831	773	4	10	8
40	0	NO_loss_NO_highcharge	698	889	826	3	11	8
40	10	-	554	721	673	3	5	6
40	10	fdr_separate	547	719	675	3	5	6
40	10	NO_loss	642	826	763	4	7	8
40	10	NO_loss_NO_highcharge	690	887	822	3	10	8
40	20	-	537	722	670	3	3	3
40	20	fdr_separate	536	721	669	4	4	3
40	20	NO_loss	583	749	702	2	3	2
40	20	NO_loss_NO_highcharge	636	808	760	3	4	3

Supplementary Table S8.

Table S7. Number of correct and incorrect unique cross links for the DSS data set by Beveridge *et al* for different score and partial score values together with specific change (either none "-"; calculating FDR on separating protein inter- and intra-crosslink (fdr_separate); excluding neutral losses peak additions (NO_loss); excluding both neutral losses and high-charged peaks (NO_loss_NO_highcharge)
Result are at FDR=1%

Score	Partial	Additional change	#Correct_1	#Correct_2	#Correct_3	#Incorrect_1	#Incorrect_2	#Incorrect_3
0	0	-	210	223	203	4	9	4
0	0	fdr_separate	207	219	202	2	12	9
0	0	NO_loss	220	234	218	5	12	10
0	0	NO_loss_NO_highcharge	221	234	218	3	9	5
0	10	-	202	216	196	2	5	6
0	10	fdr_separate	204	216	197	2	11	7
0	10	NO_loss	219	234	216	3	6	10
0	10	NO_loss_NO_highcharge	221	235	218	3	9	4
0	20	-	199	212	199	3	5	4
0	20	fdr_separate	199	212	199	4	8	5
0	20	NO_loss	209	220	205	2	4	5
0	20	NO_loss_NO_highcharge	210	222	208	3	5	3
20	0	-	205	215	197	2	7	5
20	0	fdr_separate	203	213	196	2	10	7
20	0	NO_loss	218	232	217	2	9	9
20	0	NO_loss_NO_highcharge	220	234	216	2	8	7
20	10	-	200	216	195	2	5	6
20	10	fdr_separate	198	212	191	3	8	7
20	10	NO_loss	219	233	215	2	6	8
20	10	NO_loss_NO_highcharge	220	234	216	2	7	8
20	20	-	199	212	199	3	5	4
20	20	fdr_separate	199	212	200	4	8	5
20	20	NO_loss	209	220	205	2	4	5
20	20	NO_loss_NO_highcharge	210	222	208	3	5	3
40	0	-	202	211	198	2	6	3
40	0	fdr_separate	202	211	197	2	6	3
40	0	NO_loss	215	225	210	4	9	8
40	0	NO_loss_NO_highcharge	219	229	213	3	8	8
40	10	-	200	212	195	2	5	3
40	10	fdr_separate	199	211	195	2	5	3
40	10	NO_loss	214	225	208	4	6	8
40	10	NO_loss_NO_highcharge	219	229	213	3	7	8
40	20	-	200	210	201	2	3	3
40	20	fdr_separate	200	210	201	3	4	3
40	20	NO_loss	199	213	198	2	3	2
40	20	NO_loss_NO_highcharge	205	215	201	3	4	3

Supplementary Table S9.

Table S8. Number of correct and incorrect CSMs for the DSBU data set by Beveridge *et al* for different score and partial score values together with specific change (either none "-"; calculating FDR on separating protein inter- and intra-crosslink (fdr_separate); excluding neutral losses peak additions (NO_loss); excluding both neutral losses and high-charged peaks (NO_loss_NO_highcharge)
Result are at FDR=1%

Score	Partial	Additional change	#Correct	#Incorrect
0	0	-	920	25
0	0	fdr_separate	1027	21
0	0	NO_loss	1009	18
0	0	NO_loss_NO_highcharge	1062	20
0	10	-	931	15
0	10	fdr_separate	1009	14
0	10	NO_loss	1011	13
0	10	NO_loss_NO_highcharge	1067	15
0	20	-	777	10
0	20	fdr_separate	777	5
0	20	NO_loss	889	8
0	20	NO_loss_NO_highcharge	972	8
20	0	-	940	23
20	0	fdr_separate	984	21
20	0	NO_loss	1013	17
20	0	NO_loss_NO_highcharge	1071	21
20	10	-	924	16
20	10	fdr_separate	971	9
20	10	NO_loss	1013	13
20	10	NO_loss_NO_highcharge	1081	14
20	20	-	777	10
20	20	fdr_separate	777	7
20	20	NO_loss	889	8
20	20	NO_loss_NO_highcharge	972	8
40	0	-	750	20
40	0	fdr_separate	750	16
40	0	NO_loss	861	16
40	0	NO_loss_NO_highcharge	982	19
40	10	-	747	12
40	10	fdr_separate	747	10
40	10	NO_loss	854	11
40	10	NO_loss_NO_highcharge	977	13
40	20	-	665	6
40	20	fdr_separate	665	4
40	20	NO_loss	790	8
40	20	NO_loss_NO_highcharge	914	8

Supplementary Table S10.

Table S9. Number of correct and incorrect unique cross links for the DSBU data set by Beveridge *et al* for different score and partial score values together with specific change (either none "-"; calculating FDR on separating protein inter- and intra-crosslink (fdr_separate); excluding neutral losses peak additions (NO_loss); excluding both neutral losses and high-charged peaks (NO_loss_NO_highcharge) Result are at FDR=1%

Score	Partial	Additional change	#Correct	#Incorrect
0	0	-	232	23
0	0	fdr_separate	242	19
0	0	NO_loss	239	15
0	0	NO_loss_NO_highcharge	242	18
0	10	-	231	14
0	10	fdr_separate	237	13
0	10	NO_loss	239	11
0	10	NO_loss_NO_highcharge	240	13
0	20	-	224	8
0	20	fdr_separate	224	4
0	20	NO_loss	231	6
0	20	NO_loss_NO_highcharge	236	6
20	0	-	234	22
20	0	fdr_separate	238	20
20	0	NO_loss	241	15
20	0	NO_loss_NO_highcharge	242	19
20	10	-	232	15
20	10	fdr_separate	236	8
20	10	NO_loss	239	11
20	10	NO_loss_NO_highcharge	242	12
20	20	-	224	8
20	20	fdr_separate	224	6
20	20	NO_loss	231	6
20	20	NO_loss_NO_highcharge	236	6
40	0	-	225	20
40	0	fdr_separate	225	16
40	0	NO_loss	233	13
40	0	NO_loss_NO_highcharge	239	17
40	10	-	225	12
40	10	fdr_separate	225	10
40	10	NO_loss	232	9
40	10	NO_loss_NO_highcharge	239	11
40	20	-	217	6
40	20	fdr_separate	217	4
40	20	NO_loss	225	6
40	20	NO_loss_NO_highcharge	233	6

Supplementary Table S11.

Table S10. Number of correct and incorrect CSMs for the DSSO data set by Beveridge *et al* for different score and partial score values together with specific change (either none “-“; calculating FDR on separating protein inter- and intra-crosslink (fdr_separate); excluding neutral losses peak additions (NO_loss); excluding both neutral losses and high-charged peaks (NO_loss_NO_highcharge)
Result are at FDR=1%

Score	Partial	Additional change	#Correct	#Incorrect
0	0	-	556	7
0	0	fdr_separate	878	14
0	0	NO_loss	894	16
0	0	NO_loss_NO_highcharge	951	21
0	10	-	619	8
0	10	fdr_separate	854	4
0	10	NO_loss	928	10
0	10	NO_loss_NO_highcharge	967	17
0	20	-	685	2
0	20	fdr_separate	685	2
0	20	NO_loss	781	2
0	20	NO_loss_NO_highcharge	860	2
20	0	-	458	6
20	0	fdr_separate	858	9
20	0	NO_loss	926	16
20	0	NO_loss_NO_highcharge	972	21
20	10	-	825	12
20	10	fdr_separate	827	4
20	10	NO_loss	916	10
20	10	NO_loss_NO_highcharge	966	18
20	20	-	683	2
20	20	fdr_separate	683	2
20	20	NO_loss	781	2
20	20	NO_loss_NO_highcharge	860	2
40	0	-	701	11
40	0	fdr_separate	702	8
40	0	NO_loss	819	12
40	0	NO_loss_NO_highcharge	926	22
40	10	-	691	7
40	10	fdr_separate	691	4
40	10	NO_loss	810	7
40	10	NO_loss_NO_highcharge	915	16
40	20	-	610	2
40	20	fdr_separate	610	2
40	20	NO_loss	731	2
40	20	NO_loss_NO_highcharge	835	2

Supplementary Table S12.

Table S11. Number of correct and incorrect unique cross links for the DSSO data set by Beveridge *et al* for different score and partial score values together with specific change (either none "-"; calculating FDR on separating protein inter- and intra-crosslink (fdr_separate); excluding neutral losses peak additions (NO_loss); excluding both neutral losses and high-charged peaks (NO_loss_NO_highcharge) Result are at FDR=1%

Score	Partial	Additional change	#Correct	#Incorrect
0	0	-	157	5
0	0	fdr_separate	183	11
0	0	NO_loss	183	12
0	0	NO_loss_NO_highcharge	185	11
0	10	-	165	6
0	10	fdr_separate	181	4
0	10	NO_loss	184	6
0	10	NO_loss_NO_highcharge	185	8
0	20	-	169	2
0	20	fdr_separate	169	2
0	20	NO_loss	176	2
0	20	NO_loss_NO_highcharge	180	2
20	0	-	141	4
20	0	fdr_separate	182	6
20	0	NO_loss	186	11
20	0	NO_loss_NO_highcharge	186	11
20	10	-	179	10
20	10	fdr_separate	179	4
20	10	NO_loss	184	6
20	10	NO_loss_NO_highcharge	185	9
20	20	-	169	2
20	20	fdr_separate	169	2
20	20	NO_loss	176	2
20	20	NO_loss_NO_highcharge	180	2
40	0	-	170	9
40	0	fdr_separate	170	5
40	0	NO_loss	178	10
40	0	NO_loss_NO_highcharge	184	12
40	10	-	170	7
40	10	fdr_separate	170	4
40	10	NO_loss	176	5
40	10	NO_loss_NO_highcharge	183	7
40	20	-	164	2
40	20	fdr_separate	164	2
40	20	NO_loss	172	2
40	20	NO_loss_NO_highcharge	179	2

Supplementary Table S13.

Table S12. TIMS-TOF results for the parameter screening for DSBU data set								
Score	Partial	Extra	#CSMs BSA-BSA	#CSMs Other-intras	#CSMs All-Inter	#CSMs BSA linked to other proteins	#Links	#Links (BSA-BSA)
0	0	-	221	0	2	1	120	117
0	0	NO_loss_NO_highcharge	234	0	3	5	128	120
0	0	fdr-separate	251	0	0	4	135	131
0	10	-	207	0	2	1	115	112
0	10	NO_loss_NO_highcharge	233	0	3	3	126	120
0	10	fdr-separate	243	0	0	0	127	127
0	10	NO_loss_NO_highcharge and fdr-separate	255	0	2	3	132	127
0	20	-	196	0	1	1	107	105
0	20	NO_loss_NO_highcharge	231	0	1	2	122	119
0	20	fdr-separate	211	0	0	0	111	111
20	0	-	222	0	2	2	122	118
20	0	NO_loss_NO_highcharge	231	0	3	3	125	119
20	0	fdr-separate	246	0	0	6	134	128
20	10	-	209	0	2	1	117	114
20	10	NO_loss_NO_highcharge	231	0	3	3	125	119
20	10	fdr-separate	242	0	0	0	126	126
20	20	-	196	0	1	1	107	105
20	20	NO_loss_NO_highcharge	231	0	1	2	122	119
20	20	fdr-separate	211	0	0	0	111	111
40	0	-	213	0	0	3	118	115
40	0	NO_loss_NO_highcharge	232	0	1	3	123	119
40	0	fdr-separate	233	0	0	1	124	123
40	10	-	216	0	0	1	117	116
40	10	NO_loss_NO_highcharge	232	0	1	2	122	119
40	10	fdr-separate	232	0	0	0	121	121
40	20	-	196	0	0	1	107	106
40	20	NO_loss_NO_highcharge	231	0	0	2	121	119
40	20	fdr-separate	207	0	0	0	111	111

Supplementary Table S14.

Table S12. TIMS-TOF results for the parameter screening for DSSO data set								
Score	Partial	Extra	#CSMs BSA-BSA	#CSMs Other-intras	#CSMs All-Inter	#CSMs BSA linked to other proteins	#Links	#Links (BSA-BSA)
0	0	-	222	0	1	3	132	128
0	0	NO_loss_NO_highcharge	236	0	0	5	137	132
0	0	fdr-separate	240	0	1	1	136	134
0	10	-	210	0	1	1	126	124
0	10	NO_loss_NO_highcharge	237	0	0	5	137	132
0	10	fdr-separate	234	0	0	1	132	131
0	10	NO_loss_NO_highcharge and fdr-separate	243	0	0	2	138	136
0	20	-	204	0	0	1	122	121
0	20	NO_loss_NO_highcharge	235	0	0	2	133	131
0	20	fdr-separate	205	0	0	0	121	121
20	0	-	210	0	0	2	127	125
20	0	NO_loss_NO_highcharge	236	0	0	5	137	132
20	0	fdr-separate	236	0	0	8	139	131
20	10	-	210	0	0	1	125	124
20	10	NO_loss_NO_highcharge	236	0	0	5	137	132
20	10	fdr-separate	233	0	0	1	131	130
20	20	-	204	0	0	1	122	121
20	20	NO_loss_NO_highcharge	235	0	0	2	133	131
20	20	fdr-separate	205	0	0	0	121	121
40	0	-	204	0	0	6	127	121
40	0	NO_loss_NO_highcharge	232	0	0	4	134	130
40	0	fdr-separate	222	0	0	4	130	126
40	10	-	204	0	0	2	123	121
40	10	NO_loss_NO_highcharge	233	0	0	4	134	130
40	10	fdr-separate	220	0	0	0	125	125
40	20	-	196	0	0	1	120	119
40	20	NO_loss_NO_highcharge	233	0	0	2	131	129
40	20	fdr-separate	197	0	0	0	119	119
0	0	-	222	0	1	3	132	128

d. Effect of increasing FDR to 5%

The below tables show the results at FDR=1% and 5% (separate protein intra- and inter-cross links). For DSS, DSSO and DSBU data sets, we used total-score=0, partial-score=10, min-match=3. For Beveridge and co-workers data set, we excluded higher charge and neutral losses. We also tested TIMS-TOF DSSO and DSBU data sets.

For DSS data set, there were slight increase with overall number of correct CSMs and slight increase for the incorrect CSMs (See **Supplementary Table S15)**. The number of correct unique cross links also remained the same but there was a slight increase in the number of incorrect cross links (from 6,14 and 9 increased to 11,20 and 13 for the replicate1, replicate2 and replicate 3, respectively (See **Supplementary Table S16**).

For the DSSO and DSBU data sets as well as TIMS-TOF DSSO and DSBU data sets, the results remained the same. Increasing here from FDR=0.001 to FDR=0.05 only caused to adding more decoy CSMs to the identification lists. We also observed the same for TIMS-TOF data set with MS-cleavable cross linkers.

We suggest that a user should use FDR=1%. First of all, increasing FDR mostly resulted in only increasing incorrect identifications or decoys. Second, we do not currently have any further FDR control on unique cross links but only on CSM-level (we have planned to do for the next project). Unique cross links were derived directly from the CSMs that were selected at given FDR and therefore the actual FDR value of the unique cross links is expected to be higher than the set CSM-FDR value.

Supplementary Table S15.

Table S12. Number of correct and incorrect CSMs for the DSS data set by Beveridge *et al.* at FDR=00.1 and FDR=0.05

FDR	#Correct_1	#Correct_2	#Correct_3	#Incorrect_1	#Incorrect_2	#Incorrect_3
0.01	737	940	880	6	18	11
0.05	745	950	882	11	24	15

Supplementary Table S16.

Table S13. Number of correct and incorrect unique cross links for the DSS data set by Beveridge *et al.* at FDR=00.1 and FDR=0.05

FDR	#Correct_1	#Correct_2	#Correct_3	#Incorrect_1	#Incorrect_2	#Incorrect_3
0.01	227	240	223	6	14	9
0.05	227	240	223	11	20	13

Re-analysis of PXD012546

Code can be found on ***https://github.com/cox-labs***

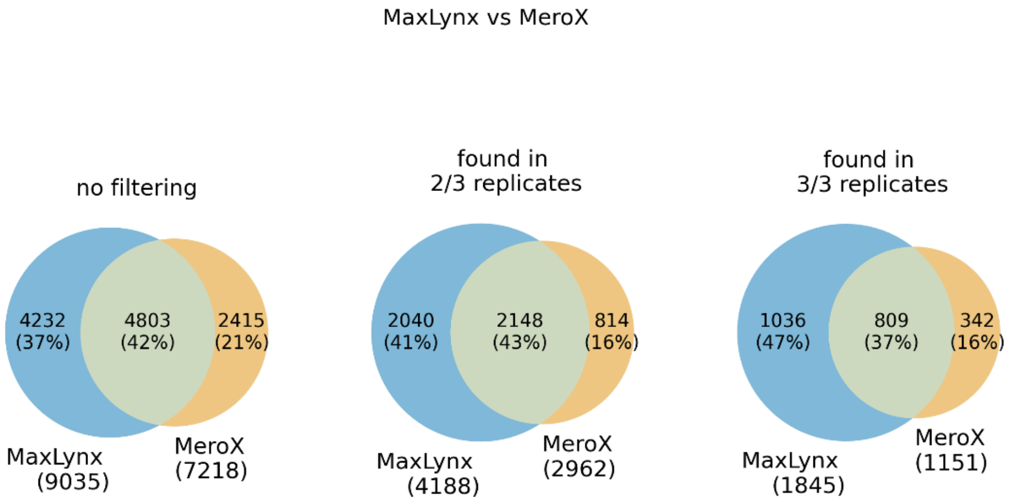

Supplementary Figure S13.
Overlap of unique cross link sites for the MaxLynx results at FDR=1% versus the MeroX results at FDR=1%

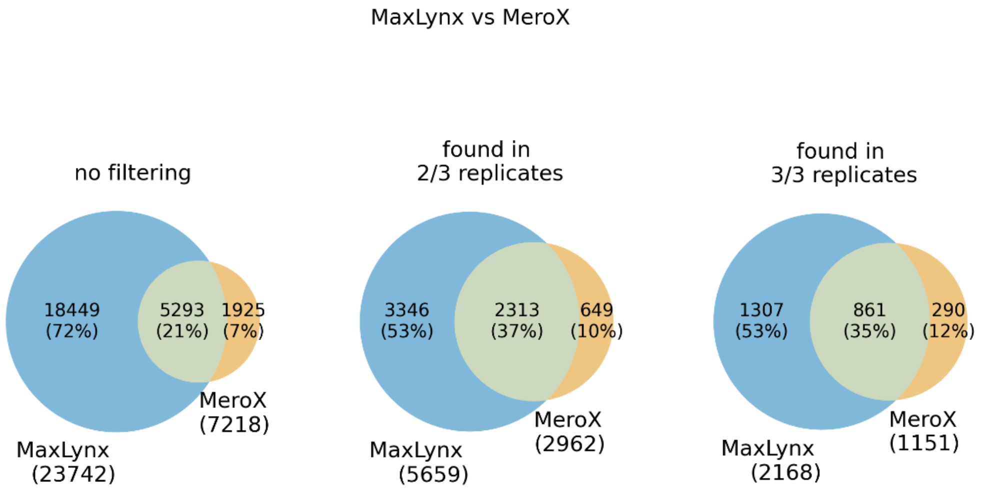

Supplementary Figure S14.
Overlap of unique cross link sites for the MaxLynx results at FDR=100% versus the MeroX results at FDR=1%

Supplementary Table S17

Table S17. Overview of CSMs and unique cross links for re-processing PXD013947 data set		
	**MaxQuant 2.0.3**	**pLink 2.3.9**
#CSMs (forward)	2542	2335
#CSMs (forward inter-protein)	410	325
#CSMs (forward intra-protein)	2132	2010
#cross links	315	287
#cross links (inter-protein)	120	94
#cross links (intra-protein)	195	193

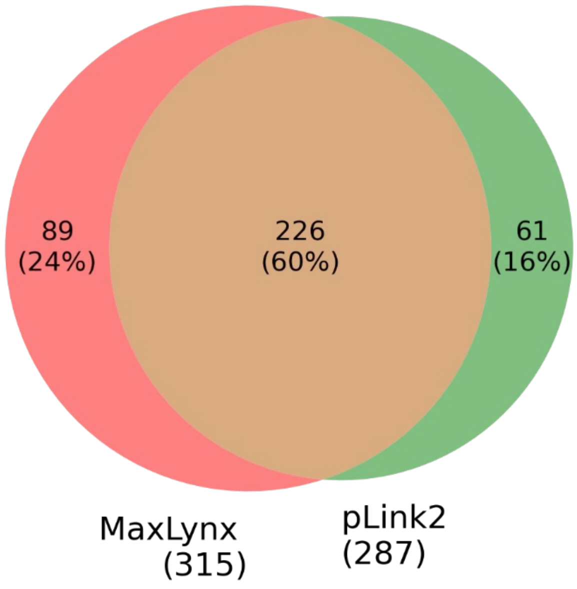

Supplementary Figure S15.

Number of unique cross links on re-analysis of PXD013947[3] The results of MaxLynx vs pLink2 at separate FDR=1%. Here we did not exclude homo-multimeric cross links.

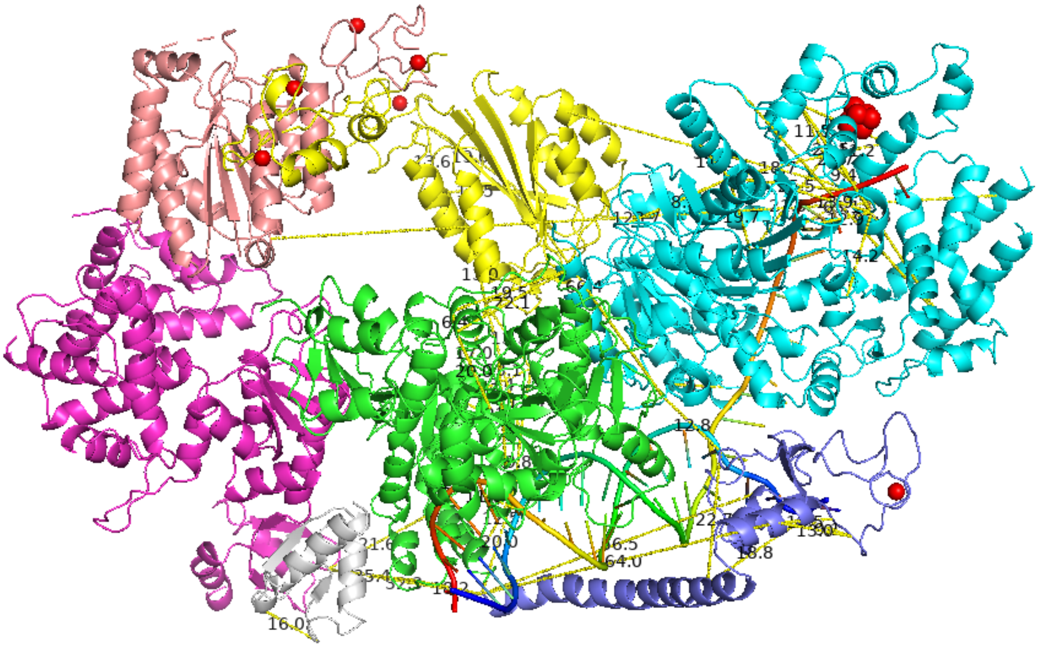

Supplementary Figure S16.

Mapped unique cross links identified by MaxLynx on re-analysis of PXD013947 on PDB 6RO4 structure.

Running times

MaxLynx run times on several benchmark data sets with different search parameter can be seen on the Supplementary Table S18.

Supplementary Table S18

Supplementary Table S18. MaxQuant complete run times for the various data sets

Cross linker	*Database size (protein entries)*	*# Thread*	*MaxLynx Step*	*Duration (min)*
**DSS (noncleavable)**	11	4	Complete MaxQuant run	32:23
			Prepare crosslink search	7:25
			MS/MS crosslink search	3:15
**TIMS-TOF DSBU (MS-cleavable)**	117	4	Complete MaxQuant run	1:30:05
			Prepare crosslink search	< 1 minute
			MS/MS crosslink search	4:41
**DSBU (MS-cleavable)**	9535	120	Complete MaxQuant run	24:13:00
			Prepare crosslink search	4:47
			MS/MS crosslink search	17:08:36

Re-analysis of PXD013947

MaxLynx was run on our Windows server (256 GB RAM, Intel® Xeon® CPU E7-4870v2), it took about two hours (The feature detection took the longest with 28 minutes. The second longest step was performing MS/MS crosslink search (about 15 minutes) and then comes preparing crosslink search with about 13 minutes (where all possible combinations were created). Because we could not run pLink2 on our Windows servers, therefore we ran pLink2 on a laptop (48 GB RAM, Intel i7-1185G7). We introduced raw files to pLink2 directly. The run time for pLink2 took about 6 minutes.

They are not directly comparable because two different machines but this can still give some idea on the usages and run time about MaxLynx for users.

References

1. Beveridge, R., Stadlmann, J., Penninger, J. M. & Mechtler, K. A synthetic peptide library for benchmarking crosslinking-mass spectrometry search engines for proteins and protein complexes. *Nat. Commun.* **11**, (2020).

2. Netz, E. *et al.* OpenPepXL: An Open-Source Tool for Sensitive Identification of Cross-Linked Peptides in XL-MS. *Mol. Cell. Proteomics* **19**, 2157–2167 (2020).

3. Kokic, G. *et al.* Structural basis of TFIIH activation for nucleotide excision repair. *Nat Comm* **10**, (2019).
